# PfSPZ-CVac efficacy against malaria increases from 0% to 75% when administered in the absence of erythrocyte stage parasitemia: A randomized, placebo-controlled trial with controlled human malaria infection

**DOI:** 10.1371/journal.ppat.1009594

**Published:** 2021-05-28

**Authors:** Sean C. Murphy, Gregory A. Deye, B. Kim Lee Sim, Shirley Galbiati, Jessie K. Kennedy, Kristen W. Cohen, Sumana Chakravarty, Natasha KC, Yonas Abebe, Eric R. James, James G. Kublin, Stephen L. Hoffman, Thomas L. Richie, Lisa A. Jackson

**Affiliations:** 1 Seattle Malaria Clinical Trials Center, Vaccine and Infectious Disease Division, Fred Hutchinson Cancer Research Center, Seattle, Washington, United States of America; 2 Department of Laboratory Medicine and Pathology, University of Washington, Seattle, Washington, United States of America; 3 Department of Microbiology, University of Washington, Seattle, Washington, United States of America; 4 Center for Emerging and Re-emerging Infectious Diseases, Seattle, Washington, United States of America; 5 Division of Microbiology and Infectious Diseases, National Institute of Allergy and Infectious Diseases, National Institutes of Health, Bethesda, Maryland, United States of America; 6 Sanaria Inc., Rockville, Maryland, United States of America; 7 The Emmes Company, Rockville, Maryland, United States of America; 8 Department of Global Health, University of Washington, Seattle, Washington, United States of America; 9 Kaiser Permanente Washington Health Research Institute, Seattle, Washington, United States of America; University of South Florida, UNITED STATES

## Abstract

PfSPZ-CVac combines ‘PfSPZ Challenge’, which consists of infectious *Plasmodium falciparum* sporozoites (PfSPZ), with concurrent antimalarial chemoprophylaxis. In a previously-published PfSPZ-CVac study, three doses of 5.12x10^4^ PfSPZ-CVac given 28 days apart had 100% vaccine efficacy (VE) against controlled human malaria infection (CHMI) 10 weeks after the last immunization, while the same dose given as three injections five days apart had 63% VE. Here, we conducted a dose escalation trial of similarly condensed schedules. Of the groups proceeding to CHMI, the first study group received three direct venous inoculations (DVIs) of a dose of 5.12x10^4^ PfSPZ-CVac seven days apart and the next full dose group received three DVIs of a higher dose of 1.024x10^5^ PfSPZ-CVac five days apart. CHMI (3.2x10^3^ PfSPZ Challenge) was performed by DVI 10 weeks after the last vaccination. In both CHMI groups, transient parasitemia occurred starting seven days after each vaccination. For the seven-day interval group, the second and third vaccinations were therefore administered coincident with parasitemia from the prior vaccination. Parasitemia was associated with systemic symptoms which were severe in 25% of subjects. VE in the seven-day group was 0% (7/7 infected) and in the higher-dose, five-day group was 75% (2/8 infected). Thus, the same dose of PfSPZ-CVac previously associated with 63% VE when given on a five-day schedule in the prior study had zero VE here when given on a seven-day schedule, while a double dose given on a five-day schedule here achieved 75% VE. The relative contributions of the five-day schedule and/or the higher dose to improved VE warrant further investigation. It is notable that administration of PfSPZ-CVac on a schedule where vaccine administration coincided with blood-stage parasitemia was associated with an absence of sterile protective immunity.

**Clinical trials registration**: NCT02773979.

## Author summary

The world needs a protective malaria vaccine. One approach is to repeatedly administer whole sporozoites, the parasite form that is transmitted from mosquitos to humans. Without treatment, sporozoites enter the liver, grow for a week, and then infect red blood cells, causing clinical disease. Here, we gave a vaccine consisting of sporozoites with a drug that prevents red blood cell infections to eliminate clinical illness. This approach was protective in other studies so we initially evaluated a faster schedule where the vaccine was given weekly. Surprisingly, there was no protection observed. We determined that weekly intervals led the second and third vaccine doses to be administered just as the previous dose of sporozoites was transitioning from the liver to the blood stage. Even though blood stage infection was stopped in this study by the co-administered drug (chloroquine), we hypothesized that it was problematic to administer a vaccine during blood stage infection. Therefore, we gave the vaccinations every five days so that upon the second and third doses, there were no blood-stage parasites present. With five-day spacing and a higher dose, the vaccine protected 75% of participants. These findings suggest that blood-stage infections may hinder formation of protective responses to malaria.

## Introduction

Development of a malaria vaccine that provides high level durable protection is a global health priority. Use of whole *Plasmodium falciparum* sporozoites (PfSPZ) is one of the approaches being pursued and is the only malaria vaccine strategy that has been shown to induce >90% sterilizing protection against controlled human malaria infection (CHMI) [1–3]. It has been known since the 1970s that radiation-attenuated whole sporozoites, which are capable of hepatocyte invasion but cannot replicate and progress to mature liver-stage schizonts, can provide high level protection against CHMI [1,4–7]. Administration of replication competent PfSPZ capable of completing and exiting the liver stage of the parasitic life cycle could induce a more potent and broader immune response than irradiated sporozoites, provided that the blood stage replication cycle can be blocked.

In an initial exploration of such an approach in the Netherlands, 10 trial subjects received fully infectious *P*. *falciparum* sporozoites, via the bites of 12 to 15 infected mosquitoes, three times at four-week intervals, while taking weekly chloroquine (CQ) doses throughout the vaccination period to interrupt blood stage replication [3]. As expected, since CQ does not affect the early ring forms of the first generation of blood-stage parasites [8], transient blood stage parasitemia was detected by a polymerase chain reaction (PCR) assay starting at seven days after each vaccination. All subjects were protected against CHMI administered eight weeks after the last vaccination and four of six (67%) were protected against repeat CHMI 26 months later [9]. The protection seen in the initial study in the Netherlands was replicated there in a second study [10] but showed diminished efficacy against sporozoite challenge in two other similarly-designed studies [11–12].

To allow practical and reproducible PfSPZ administration, Sanaria manufactures PfSPZ Challenge, consisting of infectious (replication-intact), cryopreserved, PfSPZ that are optimally administered by direct venous inoculation (DVI) [2]. The vaccine approach combining DVI of PfSPZ Challenge with concurrent administration of antimalarial chemoprophylaxis is termed PfSPZ-CVac (PfSPZ chemoprophylaxis vaccination) [2,13]. The PfSPZ Challenge product can also be administered alone at a lower dose to perform CHMI [14–16].

In a clinical trial of PfSPZ-CVac in malaria naïve adults in Germany, escalating doses of PfSPZ Challenge were administered by DVI on varying schedules under CQ prophylaxis [2]. Administration of the highest dose of 5.12x10^4^ PfSPZ of PfSPZ Challenge given as three injections 28 days apart to nine subjects conferred 100% protection against CHMI at 10 weeks after the last immunization. The study also evaluated two condensed schedules of the 5.12x10^4^ dose, three vaccinations given 14 days apart and three vaccinations given five days apart. The condensed schedules, however, conferred lower protection against CHMI, with vaccine efficacy (VE) of 67% (6/9 protected) for the 14-day schedule and 63% VE (5/8 protected) for the 5-day schedule.

As a follow-up to that study, we conducted a dose-escalation trial to evaluate whether higher doses of PfSPZ Challenge could improve VE of a condensed PfSPZ-CVac schedule. The first study group received a dose of 5.12x10^4^ PfSPZ of PfSPZ Challenge given as three injections seven days apart, with the intent to progress to higher doses given on the same schedule in subsequent cohorts. The seven-day interval was chosen for logistical ease. During the trial, the safety and efficacy profile of the first group necessitated changing the study design (as fully described in Methods) to evaluate a group administered three injections of 1.024x10^5^ PfSPZ five days apart. Here, we present the safety, efficacy, and immunogenicity results as well as patterns of blood stage parasitemia following vaccination and CHMI.

## Methods

### Ethics statement

The study and all revisions were reviewed and approved by the Quorum Review U.S. Institutional Review Board (Panel I) as Protocol #11–0042. Written informed consent was obtained from all participants for all study procedures.

### Participants

Eligible participants were non-pregnant malaria-naïve healthy adults 18 through 45 years of age. Complete eligibility criteria are listed at clinicaltrials.gov (NCT02773979).

### Study design—Initial and revised approaches

The trial was designed as a Phase 1, randomized, placebo controlled, dose escalation study, with PfSPZ Challenge or normal saline placebo administered by DVI in a three-dose schedule given seven days apart. Dose escalation was to progress by two-fold increases from 5.12x10^4^ PfSPZ of PfSPZ Challenge per injection in Group 1 to 1.024x10^5^ PfSPZ in Group 2 to 2.048x10^5^ PfSPZ in Group 3. Each group was to include 12 subjects, randomized 3:1 to receive PfSPZ Challenge or placebo (randomization and blinding details in S1 Text). All subjects were to receive four weekly doses of oral CQ during the vaccination phase (Table 1). Ten weeks after the last vaccination, enough time for CQ concentration to fall to inactive levels, homologous CHMI was to be administered to each group by DVI of 3.2x10^3^ PfSPZ of PfSPZ Challenge.

**Table 1:** Study groups, schedules, study products, and vaccine efficacy.

Group	Chloroquine dosing	Vaccine dosing	PfSPZ or control product	CHMI^1^	Vaccine efficacy
1	Days 1, 8, 15, 22	Days 3, 10, 17	5.12x10^4^ PfSPZ (n = 9)^2^ or saline placebo (n = 3)	10 weeks after 3^rd^ vaccination^3^	0% (0/7 protected)^4^
2	Days 1, 8, 15, 22	Days 3, 10, 17	1.024x10^5^ PfSPZ (n = 3) or saline placebo (n = 1)	Not performed	N/A
3	Days 1, 6, 11, 16	Days 1, 6, 11	1.024x10^5^ PfSPZ (n = 9)^5^	10 weeks after 3^rd^ vaccination^6^	75% (6/8 protected)^7^

DVI = direct venous inoculation; CHMI = controlled human malaria infection

^1^PfSPZ Challenge dose of 3.2x10^3^ PfSPZ by DVI.

^2^Two PfSPZ Challenge recipients were discontinued from vaccinations and did not undergo CHMI.

^3^Treatment definition for Group 1 CHMI: positive qRT-PCR with a parasite density of ≥250 parasites/mL within 28 days post-CHMI confirmed by a positive qRT-PCR (of any density) from another sample collected 6–60 hours before or after the index sample.

^4^Three of three placebo control participants developed parasitemia post-CHMI.

^5^One PfSPZ Challenge recipient was discontinued from vaccinations and did not undergo CHMI.

^6^Treatment definition for Group 3 CHMI: one positive qRT-PCR with a parasite density of ≥20 parasites/mL within 28 days post-CHMI.

^7^Three of three non-immunized infectivity control participants developed parasitemia post-CHMI.

In Group 1, solicited systemic adverse events (AEs) were common after PfSPZ Challenge administration and one subject was discontinued after the second injection due to grade 3 AEs associated with parasitemia from the first PfSPZ Challenge dose. The Safety Monitoring Committee (SMC) therefore recommended that escalation to Group 2 should start with evaluation of only four subjects (three assigned to vaccine, one to placebo).

Prior to the planned safety review of the Group 2 pilot, the CHMI for Group 1 was completed. All three placebo controls and seven vaccinated subjects became infected, for a VE of 0%. Based on this information, the investigators requested, and the SMC approved, a protocol amendment to change the dose schedule for a newly defined Group 3 to three doses given five days apart, to allow direct comparison with the prior study in Germany [2], which had studied the five day schedule, while maintaining the same (rather than escalating) PfSPZ Challenge dose used to immunize the Group 2 pilot. Normal saline controls were removed from Group 3, as blinded treatment assignment in Group 1 had proven futile due to the increased frequency of AEs during intervals of parasitemia in the active group, and were replaced with three infectivity controls enrolled just prior to CHMI. To allow earlier initiation of treatment after onset of parasitemia, the treatment definition applied to the CHMI phase was modified as specified in Table 1.

### Objectives

The primary objective was to evaluate the safety and tolerability of escalating doses of Sanaria PfSPZ Challenge administered by DVI on varying schedules to malaria-naïve adults taking prophylactic doses of CQ (PfSPZ-CVac). Exploratory objectives included evaluating the efficacy of PfSPZ-CVac against CHMI, assessing the occurrence and density of blood stage parasitemia during the second week following doses of PfSPZ Challenge, assessing humoral and cellular immune responses to PfSPZ-CVac, and assessing the pharmacokinetic profile of CQ.

### Safety monitoring

Safety was monitored by identification of serious adverse events from enrollment through the end of study follow-up at 57 days after CHMI. During the vaccination phase, solicited systemic AEs were recorded from the day of enrollment through 14 days after the third DVI, solicited injection site AEs were recorded for seven days after each DVI, and unsolicited AEs were recorded from enrollment through 14 days after the third DVI. After CHMI, solicited systemic AEs were recorded for 29 days, solicited local AEs were recorded for six days, and unsolicited AEs were recorded for 43 days.

Due to rare reported cardiac events post-CHMI in other studies [17,18], subjects were asked at each visit whether they had experienced cardiovascular symptoms and positive responses were further evaluated. Clinical laboratory evaluations for safety (alkaline phosphatase, alanine aminotransferase, aspartate aminotransferase, total bilirubin, creatinine, glucose, potassium, white blood cell count, hemoglobin and platelet count) were performed five days after each study vaccination, prior to and one, three, and 23 days after CHMI administration, and after the treatment definition for blood stage parasitemia was met (if applicable).

### Study products

Sanaria PfSPZ Challenge, composed of aseptic, purified, cryopreserved, infectious NF54 strain PfSPZ, was supplied in vials containing 1.5x10^4^ PfSPZ for CHMI or 1.0x10^5^ PfSPZ for immunizations in 20 μL and stored in liquid nitrogen vapor phase [2,15]. Vials were thawed and constituted in phosphate buffered saline containing 1% human serum albumin to 0.5 mL in a 1 mL syringe and injected by DVI into the antecubital or other arm vein using a 25G needle. Placebo consisted of 0.5 mL of 0.9% sodium chloride, USP (normal saline) loaded and injected using identical syringes. Two 500 mg tablets of chloroquine phosphate (Rising Pharmaceuticals, East Brunswick, NJ), each containing 300 mg base, were given for the first dose and one 500 mg tablet was given for three subsequent doses.

### Assays

#### *Plasmodium* 18S rRNA reverse transcription PCR (qRT-PCR)

The qRT-PCR assay [19,20] detects and quantifies *Plasmodium* 18S rRNA and reports results as estimated parasites/mL of blood (reportable range 20 parasites/mL to >4x10^8^ parasites/mL). During the vaccination phase, blood specimens for Groups 1 and 2, which were immunized on study days 3, 10 and 17, were collected for qRT-PCR testing on study days 8–20, 22–27, 31 and 45. For Group 3, which was immunized on study days 1, 6 and 11, blood specimens were collected on study days 6–21, 25 and 39. In the CHMI phase, blood specimens were collected on days 7–21, 23, 25 and 29 post-CHMI.

#### Humoral assays

Sera were assessed for anti-Pf circumsporozoite protein (CSP) IgG antibody levels by an enzyme-linked immunosorbent assay (ELISA) using recombinant PfCSP, for antibody responses against whole PfSPZ by an automated immunofluorescence (aIFA) assay, and for levels of functional antibodies by an automated inhibition of sporozoite invasion (aISI) assay. All assays were performed by Sanaria, as previously described [2,21,22].

#### T-cell assays

Cellular immune responses were assessed using intracellular cytokine staining (ICS) and multiparameter flow cytometry of cryopreserved peripheral blood mononuclear cells (PBMCs) as described [23–25] with the following modifications. PBMCs (1x10^6^) were thawed and stained with a multiparameter flow cytometry panel to evaluate the phenotype and cell population frequencies including TCR-γδ, TCR-Vδ2, and TCR-Vγ9. The remaining PBMCs were rested for 6–8 hours at 37°C and then stimulated for 12 hours with irradiated PfSPZ (Sanaria) or with Pf-infected red blood cells (iRBCs; Sanaria). After 12 hours of stimulation, brefeldin A and monensin were added to block cytokine secretion and to allow for cytokine accumulation. After incubation for an additional five hours, cells were stained for viability and with surface markers including TCR-γδ. ICS was then performed to identify the T cell lineages (CD3, CD4 and CD8), and cytokines produced. Two different cytokine combinations—interferon-γ (IFN-γ), interleukin-2 (IL2) and/or tumor necrosis factor α (TNF-α) (subset one), and IFN-γ, IL2 and/or CD154 (subset two)—were analyzed.

#### Plasma CQ concentrations

CQ concentrations in samples obtained two days after each dose were measured by high performance liquid chromatography/tandem mass spectrometry by NMS Labs (Willow Grove, PA).

### Statistical analysis

#### Efficacy

Kaplan-Meier survival curves were generated for time to first qRT-PCR positive for each vaccine and control group. Differences in the distributions of time to first qRT-PCR positive between vaccinated and control groups were evaluated using the log-rank test conducted in a permutation test framework.

#### Immunogenicity

For the humoral immunogenicity markers, definitions for a positive response were taken relative to the pre-dose 1 measurement. For ELISA, samples were considered positive if the difference between the post-immunization optical density (OD) 1.0 and the pre-immunization OD 1.0 (net OD 1.0) was ≥50 and the ratio of the post-immunization OD 1.0 to pre-immunization OD 1.0 (ratio) was ≥3.0. For aIFA, subjects with net arbitrary fluorescence units (AFU) 2x10^5^ of ≥150 and ratio AFU 2x10^5^ of ≥3.0 were considered positive. For aISI, subjects with net ISI reciprocal serum dilution for 80% inhibition of ≥10 and ratio ISI reciprocal serum dilution for 80% inhibition of ≥3.0 were considered positive.

For cellular immunogenicity markers, percentages of PBMC subsets secreting specific cytokines on stimulation with malaria antigens were adjusted for background response by subtracting the percentage of cytokine positive cells in the control well from the percentage of cytokine positive cells in the antigen-stimulated well. The corresponding control stimulations for iRBC and PfSPZ were uninfected RBCs and human serum albumin, respectively. To identify T cell responses, the Mixture Models for Single-Cell Assays (MIMOSA) method was used to identify subjects in each treatment group with positive cytokine responses [26].

#### CQ pharmacokinetics

The relationship between the CQ levels obtained two days after CQ doses 2, 3, and 4 with the peak parasite density (PPD) observed in the corresponding 7- to 10- days following vaccinations 1, 2, and 3, respectively, was explored using a linear mixed modeling approach. Since the mixed model assumes normally distributed errors, a square root transformation was applied to PPD. The model was fit with first-order autoregressive correlation structure and included fixed effects of CQ level, group, vaccination number, and group-by-vaccination-number interaction.

## Results

The evolution of this study is briefly described below because the outcomes of the early cohorts necessitated protocol revisions mid-study. The study was conducted at the Kaiser Permanente Washington Health Research Institute in Seattle, WA between 12 September 2016 (site activation) and 22 January 2018 (last participant visit). The study was originally designed to utilize randomized, placebo-controlled, dose-escalation groups of healthy, malaria-naïve adults (9 vaccinees and 3 placebo recipients per group) to test the safety, immunogenicity, and efficacy of an experimental malaria vaccine consisting of PfSPZ Challenge administered by DVI in a three-dose schedule with concurrent oral CQ treatment during the vaccination phase, with target doses of 5.12x10^4^, 1.024x10^5^ and 2.048x10^5^ PfSPZ per injection in the three groups. To enable rapid vaccination, the vaccine doses were to be given seven days apart. This was the case for Groups 1 and 2, but the protocol revisions mid-study due to a lack of VE in Group 1, and the adverse event profile in Groups 1 and 2 revealing the identity of placebo recipients, led us to administer the Group 3 vaccinations five days apart using the same dosages as in Group 2 and without a placebo group to determine if this restored VE. Additional details about the Group 1 and 2 outcomes are described in the *Results* sub-sections below. CHMI with the homologous parasite strain (same PfNF54 strain as in the vaccine) was conducted in Groups 1 and 3 at 10 weeks post-vaccination after complete CQ clearance. Normal saline placebo immunized study subjects served as infectivity controls for Group 1 CHMI, whereas newly enrolled, non-immunized infectivity control participants were used for Group 3 CHMI. The earlier study in Germany using the same CHMI methods showed that 13/13 saline placebo control participants who received CQ and were challenged 8–10 weeks later all developed blood stage parasitemia [2], making the use of CQ-treated placebo controls unnecessary for this study. Detailed study information is shown in Table 1 and in the Methods section.

**Fig 1:**
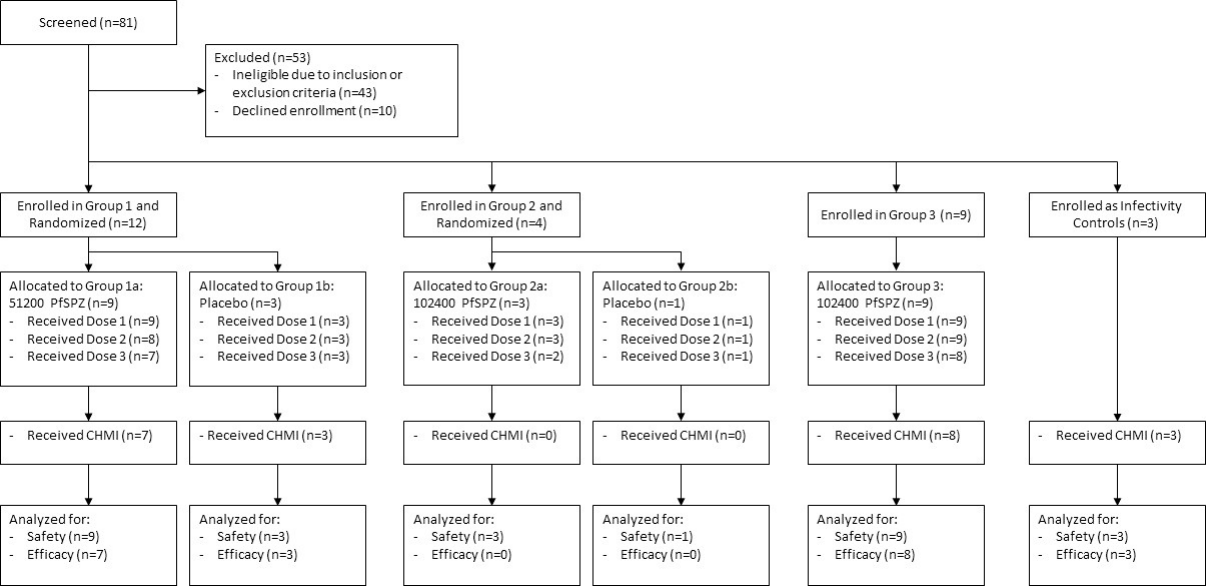
Consort flowchart.

The disposition of study subjects is shown in Fig 1. One subject was discharged from Group 1 after the first PfSPZ Challenge DVI due to poor compliance with study visits and was presumptively treated with atovaquone/proguanil. In each of Groups 1, 2, and 3, one subject was discontinued from receipt of the third PfSPZ Challenge DVI due to vaccine-related AEs. The demographic characteristics of study subjects were comparable across groups (S1 Table).

### Tolerability and local and systemic AEs post-vaccine administration

The administration of PfSPZ was very well tolerated as measured by the frequency of AEs days 1–6 after immunization (S2 Table). Solicited local reactogenicity after PfSPZ Challenge was reported by 7 of 9 (78%), 1 of 3 (33%), and 5 of 9 (56%) of participants after any dose, in Groups 1, 2, and 3, respectively, and by 2 of 4 (50%) placebo recipients. All were grade 1 in severity and consisted of bruising, induration, pain and/or tenderness at the injection site.

**Fig 2:**
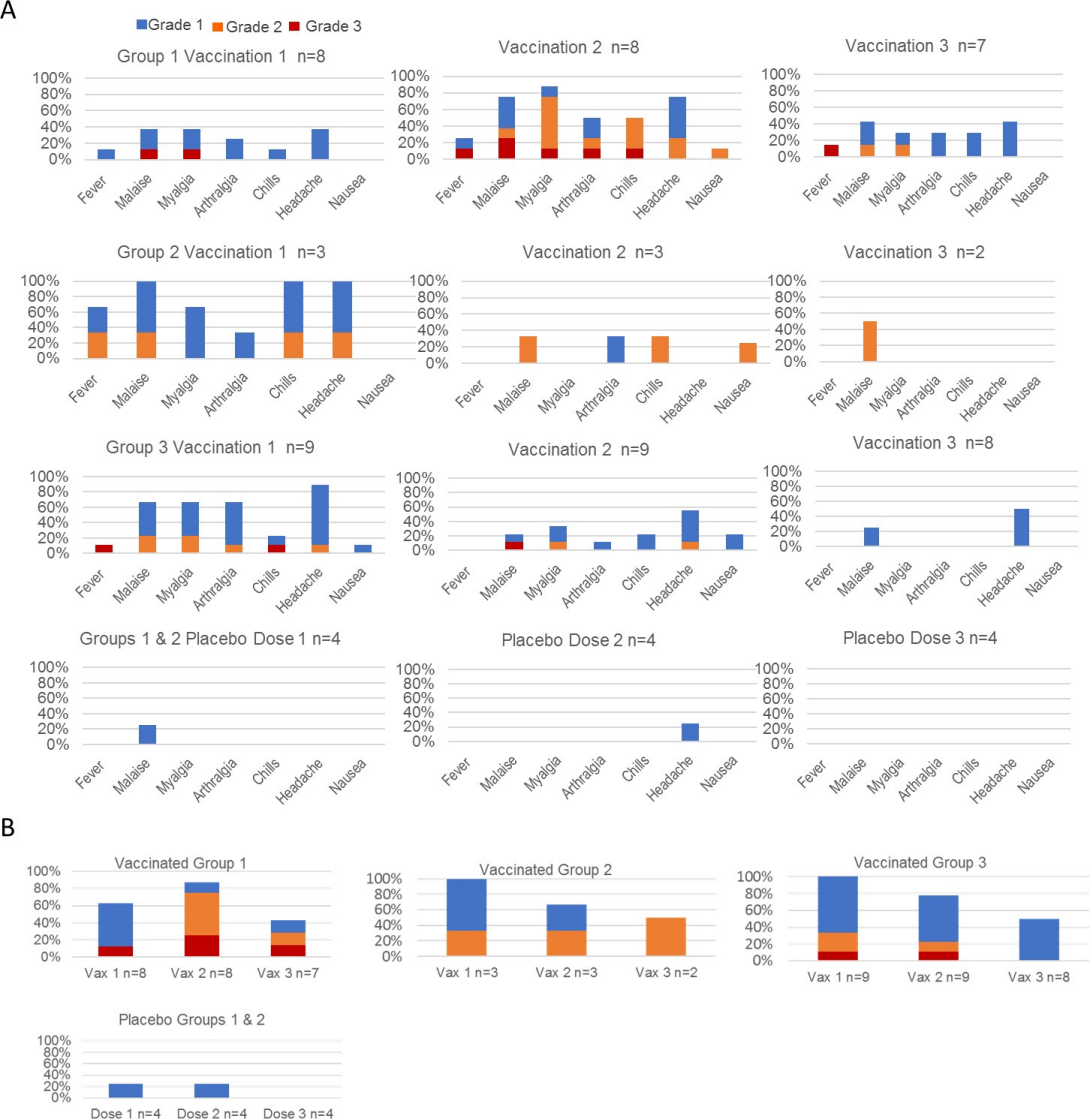
Vaccination phase AEs in vaccinated and placebo control participants. (**A**) Specific solicited systemic AEs shown as the proportion of subjects who reported a maximum grade of 3, 2, or 1 in the 7–10 days after each vaccination by group and vaccination status. (**B**) The proportion of participants in each group and amongst placebo controls who reported at least one Grade 3, Grade 2 (Grade 3 excluded), or Grade 1 (Grades 2–3 excluded) event by dose. Grade 1 events are those that required minimal or no treatment and did not interfere with daily activities. Grade 2 events are those that resulted in a low level of inconvenience or required therapeutic measures and may have interfered with functioning and daily activities. Grade 3 events are those that interrupted the subject’s usual daily activities. Temperature values are noted only if ≥38.0°C (lower limit of graded fever) and are reported as Grade 1 (38.0°C—38.4°C), Grade 2 (38.5°C—38.9°C), or Grade 3 (>38.9°C). Vomiting was a solicited systemic adverse but was not reported by any subject in the 7- to 10- days after a vaccination and so is not represented in the graphs. Blue fill (top), Grade 1; orange fill (middle), Grade 2; red fill (bottom), Grade 3.

Solicited systemic AEs were frequently reported during the intervals of parasitemia in vaccinated subjects but were infrequently reported in the 7- to 10- days after placebo injection (Fig 2A and 2B). The patterns of reported solicited systemic AEs in the 7- to 10- days after the first and second PfSPZ Challenge vaccinations were generally similar across the three groups. There appeared to be a reduction in the number and severity of AEs after the third vaccination in Groups 2 and 3 (S3 and S4 Tables). Of the 20 subjects who received at least two PfSPZ Challenge vaccinations, all reported at least one solicited systemic AE, 14 (70%) reported at least one Grade 2 or higher solicited systemic AE, and 5 (25%) reported at least one Grade 3 solicited systemic AE during the 7 to 10 days after vaccination. Unsolicited AEs judged related to vaccination were also frequently reported during periods of parasitemia and included three reports of AEs potentially referable to the cardiovascular system (S3 Table, subjects 9, 14 and 18). The results of further evaluations did not identify evidence of a cardiovascular etiology for any of these events. One additional Group 1 subject (S3 Table, subject 5), with a history of hives due to sulfa drugs and amoxicillin, reported the onset of multifocal hives five days after receiving the third vaccination that resolved within two days with oral diphenhydramine and topical hydrocortisone cream.

Safety laboratory AEs were infrequent and low grade as follows. Grade 1 AEs included elevated alanine aminotransferase (n = 3 subjects), elevated total bilirubin (n = 2 subjects), elevated potassium (n = 1 subject), elevated platelet count (n = 1 subject), decreased hemoglobin (n = 1 subject) and decreased white blood cell count (n = 3 subjects). One subject had a Grade 2 decrease in hemoglobin, and there were no Grade 3 laboratory AEs. No serious adverse events were identified.

### Blood stage parasitemia post-vaccine administration

**Fig 3:**
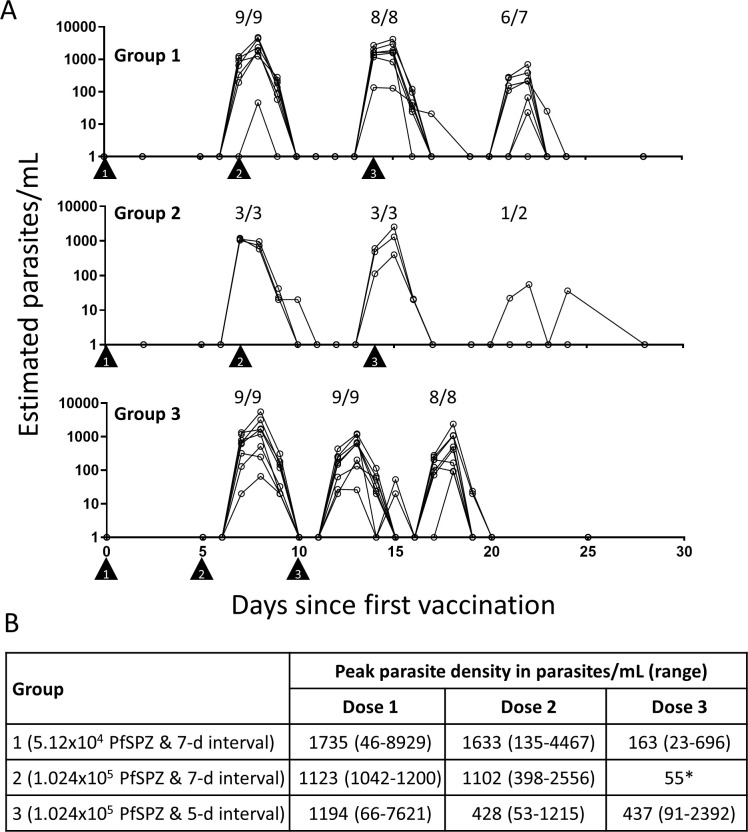
Parasite density estimated by qRT-PCR in vaccinated subjects after each vaccination by group. **(A)** Triangle symbols indicate the first, second, and third days of vaccine administration as shown. The numbers above each peak of parasitemia display the number of persons positive divided by the total number of vaccine recipients for that dose of vaccine. Days are listed relative to the first dose of vaccine. The number of participants completing each vaccination are listed above each peak of parasitemia. Group 1 received a PfSPZ Challenge dose of 5.12x10^4^ PfSPZ for each of three doses given by DVI seven days apart. Group 2 received a PfSPZ Challenge dose of 1.024x10^5^ PfSPZ for each of three doses given by DVI seven days apart. Group 3 received a PfSPZ Challenge dose of 1.024x10^5^ PfSPZ for each of three doses given by DVI five days apart. **(B)** Inset table indicates the geometric mean peak parasite density and the minimum/maximum ranges for each post-vaccination interval in estimated parasites/mL. *The value for the single subject with parasitemia.

All vaccinated subjects had transient parasitemia detected by qRT-PCR in the 7- to 10- day interval after the first and second PfSPZ Challenge doses, and most had parasitemia after the third dose (Fig 3 and S3 Table). In each interval, parasitemia persisted for three or, less commonly, four days. With this timing, the second and third vaccinations for Group 1 were administered during the period of parasitemia, but this was not the case for Group 3. For Groups 1 and 2, the geometric mean peak parasite densities (PPDs) were similar following the first and second vaccinations and declined after the third vaccination (1735, 1633, and 163 estimated parasites/mL for Group 1 and 1123, 1102, and 55 estimated parasites/mL for Group 2); the Group 1 reduction in PPD after the third dose compared to the second dose was statistically significant (p = 0.02, paired Student’s t-test). For Group 3, the geometric mean PPD was highest after the first vaccination (1194 parasites/mL), declined after the second vaccination (426 parasites/mL), and was similar following the third (437 parasites/mL) vaccination; the Group 3 reduction in PPD after the second dose compared to the first dose was borderline significant (p = 0.056, paired Student’s t-test). Parasite densities following the first vaccination were similar across the groups and did not appear to be dose dependent.

### VE against CHMI

**Fig 4:**
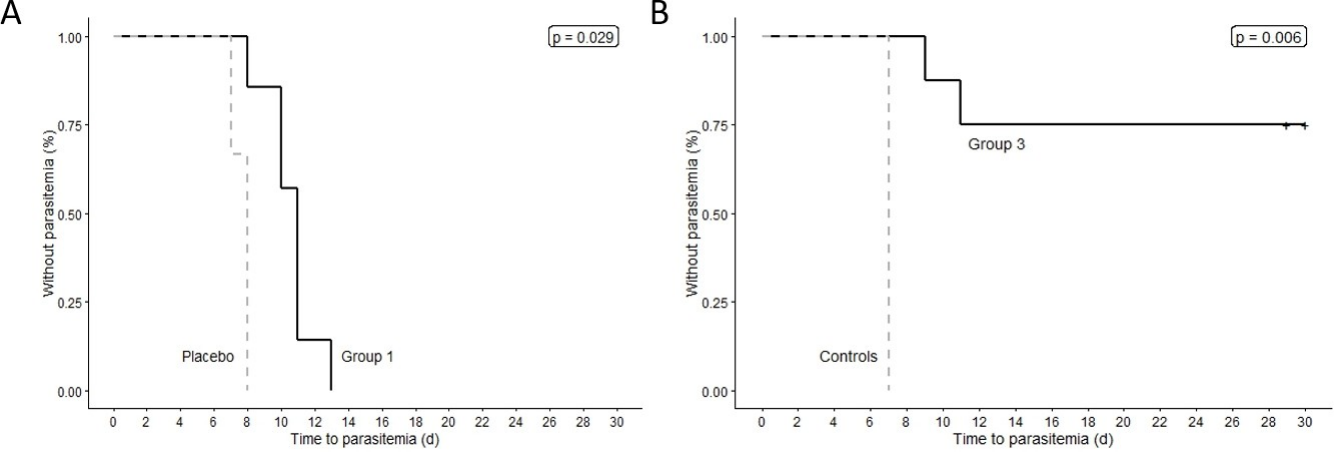
Kaplan-Meier curves for time to first *P*. *falciparum* qRT-PCR positive after CHMI in vaccinated subjects versus placebo or control subjects in Groups 1 and 3. (**A**) Group 1 (seven-day interval vaccine cohort, n = 9) and control (n = 3) data. (**B**) Group 3 (five-day interval vaccine cohort, n = 9) and control (n = 3) data. qRT-PCR limit of detection, 20 estimated parasites/mL. p values calculated using log-rank test.

All seven vaccinated subjects in Group 1 and all three placebo recipients who underwent CHMI were infected, for a VE of 0% (95% confidence interval [CI] of proportion protected: 0% to 35.4%). For Group 3, all three infectivity controls and two of the eight vaccinated subjects who underwent CHMI were infected, for a VE of 75% (95% CI of proportion protected: 40.9% to 92.8%). The onset of blood stage parasitemia (time to qRT-PCR-detected parasitemia) was significantly delayed in the seven vaccinated Group 1 subjects (days 8 to 13 post-CHMI) compared to the respective control group (day 7 post-CHMI; p = 0.029), and also in the two vaccinated Group 3 subjects who were infected (days 9 and 11 post-CHMI) compared with the respective control group (day 7 post-CHMI) (p = 0.006) (Fig 4). For subjects who were infected after CHMI, the median time to first positive qRT-PCR was 11 days for Group 1 vaccinated subjects, 8 days for Group 1 placebo subjects, 10 days for Group 3 vaccinated subjects, and 7 days for Group 3 infectivity controls. We did not see any association between parasite densities following any of the three immunizations and subsequent protection in Group 3.

### Effect of the CHMI treatment definition on patterns of parasitemia and adverse events

All subjects developing parasitemia following CHMI were treated with atovaquone/proguanil after infection detection by 18S rRNA biomarker qRT-PCR. The treatment definition applied to Group 1 (two positive RT-PCRs including one >250 estimated parasites/mL) led to later treatment following onset of parasitemia post-CHMI compared with the treatment definition applied to Group 3 (one positive qRT-PCR of ≥20 estimated parasites/mL; see S4 Table). Among the placebo subjects in Group 1, biomarker evidence of parasitemia was detected seven or eight days after CHMI administration but the treatment threshold of ≥250 estimated parasites/mL was not crossed for another three days. All placebo subjects in the Group 1 had a PPD >10000 parasites/mL, the duration of parasitemia from first detection of a positive signal (even if not yet meeting the treatment threshold) to the last detection of a positive signal post-treatment was 7–9 days, and their geometric mean PPD was 15,604 parasites/mL (95% CI; 5635–43214). In Group 3, treatment was initiated on the day after the first positive result of ≥20 estimated parasites/mL, which reduced the rate and severity of AEs: all infectivity controls had a PPD <500 parasites/mL, the duration of parasitemia was 2–4 days, and the geometric mean PPD was only 217 parasites/mL (95% CI; 36–1290). The number and severity of solicited systemic AEs during the period of parasitemia was higher in Group 1 than Group 3 (S4 Table). Even with the lower treatment threshold, all treated Group 3 participants displayed two or more days of qRT-PCR biomarker positivity. There was also no difference in the time from CHMI to first positive qRT-PCR between groups.

### Humoral immunogenicity responses

Vaccination induced humoral responses in nearly all subjects. At 14 days post dose 3, all Group 1 and Group 3 vaccinated subjects met the positive threshold for anti-PfCSP antibodies by ELISA; 6 of 7 Group 1 and 8 of 8 Group 3 subjects were anti-PfSPZ responders by IFA; and 7 of 7 Group 1 and 6 of 8 Group 3 subjects were ISI responders. At 14 days post dose 3, none of the placebo recipients were responders for any of the assays.

**Fig 5:**
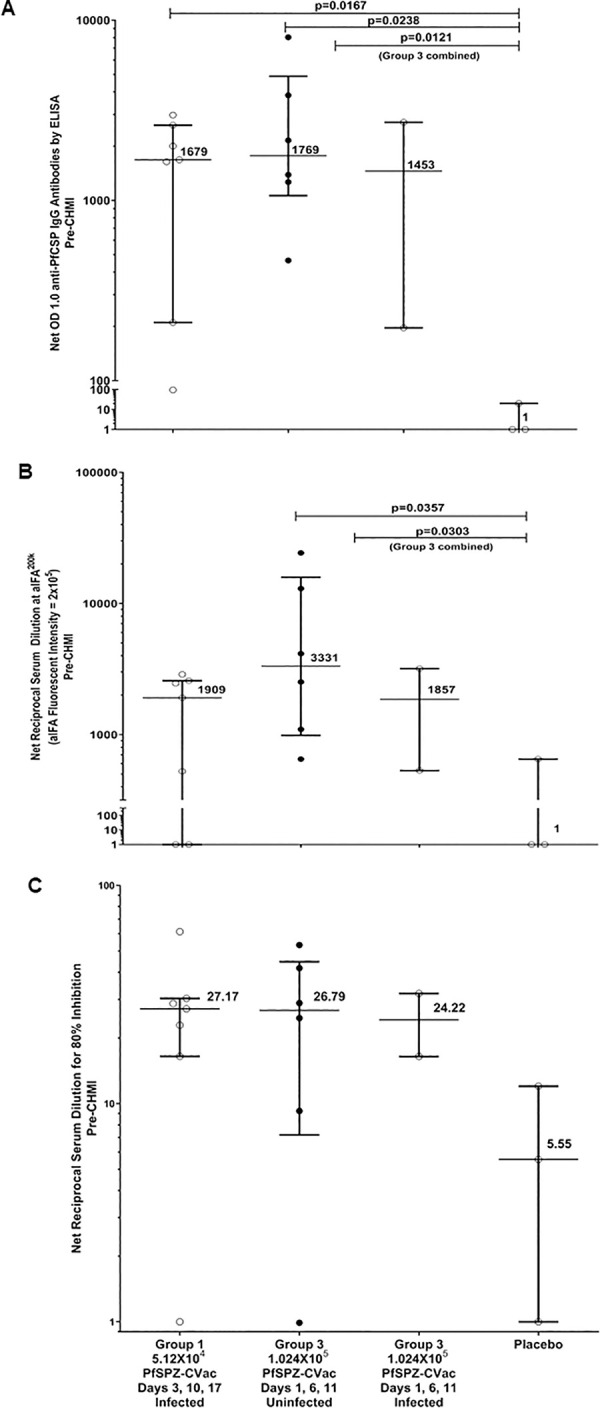
Pre-CHMI antibody comparisons between vaccination and placebo groups. (**A**) Antibodies to PfCSP (net OD 1.0) prior to CHMI. Filled circles are uninfected (protected) subjects and open circles are infected subjects. (**B**) Antibodies to PfSPZ (net aIFA^200K^) measure prior to CHMI. The y-axis refers to aIFA fluorescence intensity of 2x10^5^. Filled circles are uninfected (protected) subjects and open circles are infected subjects. (**C**) Net reciprocal serum dilution for 80% inhibition of PfSPZ invasion of hepatocytes (HC-04 cells) prior to CHMI. Filled circles are uninfected (protected) subjects and open circles are infected subjects. Horizontal lines represent medians and bars are interquartile ranges for all panels. *p* values for Wilcoxon-Mann-Whitney tests are shown in A-B.

Antibody responses in vaccinated subjects peaked at 14 days post dose 3, declined substantially by the pre-CHMI time point, and generally did not increase following CHMI (Table 2 and S1–S3 Figs). At 14 days post dose 3 and at the pre-CHMI time point, the median levels were higher in vaccinated Group 3 subjects overall than in Group 1 for anti-PfCSP and anti-PfSPZ antibodies, although differences did not achieve statistical significance (Figs 5 and S4–S6). Among Group 3 vaccinated subjects, the levels of anti-PfCSP and anti-PfSPZ antibodies at 14 days post-dose 3 and at the pre-CHMI time point were two to five times higher among two subjects infected after CHMI compared with the six subjects protected after CHMI, but the CIs were wide and overlapping and the differences were not statistically significant. The ISI assay did not show any differences between Groups 1 and 3 at any time points or between protected and non-protected subjects in Group 3. Evaluations of additive change in responses at the Day 14 post-dose 3 time point relative to baseline for each of the assays also did not discriminate between protected and unprotected subjects (Fig 5). Complete antibody response data is provided in S5 Table.

**Table 2:** Median with lower and upper quartile of humoral immunogenicity responses among subjects in Groups 1 and 3 who received three doses of PfSPZ Challenge and those who received three doses of placebo by time point and CHMI infection status.

Assay	Time point	Subjects given PfSPZ Challenge				Placebo
		Group 1 all n = 7*	Group 3 all n = 8	Group 3 infected post-CHMI n = 2	Group 3 uninfected post-CHMI n = 6	Group 1 all n = 4*
**Anti-PfCSP ELISA**	Pre-dose 1	81 (56, 116)	25 (4, 51)	20 (12, 28)	30 (1, 65)	81 (74, 87)
	14 days post-dose 3	4153 (614, 6750)	4835 (2317, 11011)	9916 (2648, 17183)	4835 (1838, 9606)	86 (22, 99)
	Pre-CHMI	1760 (266, 2750)	1844 (674, 3572)	1473 (208, 2737)	1844 (1091, 4898)	56 (1, 109)
	28 days post CHMI	1412 (630, 2269)	1529 (699, 2137)	1303 (575, 2030)	1529 (889, 2764)	197 (73, 286)
**Anti-PfSPZ IFA**	Pre-dose 1	77 (51, 510)	262 (70, 1621)	470 (77, 863)	262 (65, 1904)	375 (114, 1563)
	14 days post-dose 3	6509 (1419, 20376)	18848 (11277, 40481)	41837 (11014, 72659)	18848 (10687, 28949)	71 (43, 232)
	Pre-CHMI	1960 (565, 2872)	3736 (1706, 11075)	2327 (1394, 3260)	4302 (2269, 16106)	257 (31, 741)
	28 days post CHMI	1607 (879, 2951)	2005 (1091, 3242)	1745 (1279, 2210)	2098 (932, 4123)	172 (130, 1314)
**ISI**	Pre-dose 1	4.53 (2.92, 6.28)	6.78 (1.80, 9.62)	8.23 (6.57, 9.89)	5.60 (1.00, 10.58)	6.36 (1.00, 13.06)
	14 days post-dose 3	32.21 (22.92, 75.26)	53.06 (32.06, 77.61)	67.81 (55.26, 80.36)	46.60 (26.68, 76.48)	4.66 (1.00, 16.59)
	Pre-CHMI	33.45 (19.39, 39.32)	28.13 (19.11, 44.14)	32.45 (26.35, 38.54)	27.79 (16.75, 50.04)	6.55 (1.00, 13.02)
	28 days post CHMI	36.91 (26.73, 52.53)	29.30 (17.39, 35.05)	32.76 (30.11, 35.40)	25.84 (14.57, 41.14)	15.95 (14.12, 31.15)

Anti-PfCSP ELISA = median serum dilution at which the optical density was 1.0.

Anti-PfSPZ IFA = median serum dilution at which the arbitrary fluorescence units were 2.0x105.

ISI = median serum dilution at which there was 80% inhibition.

*All Group 1 vaccinated subjects and placebo subjects were infected after CHMI.

### Cellular immunogenicity

**Fig 6:**
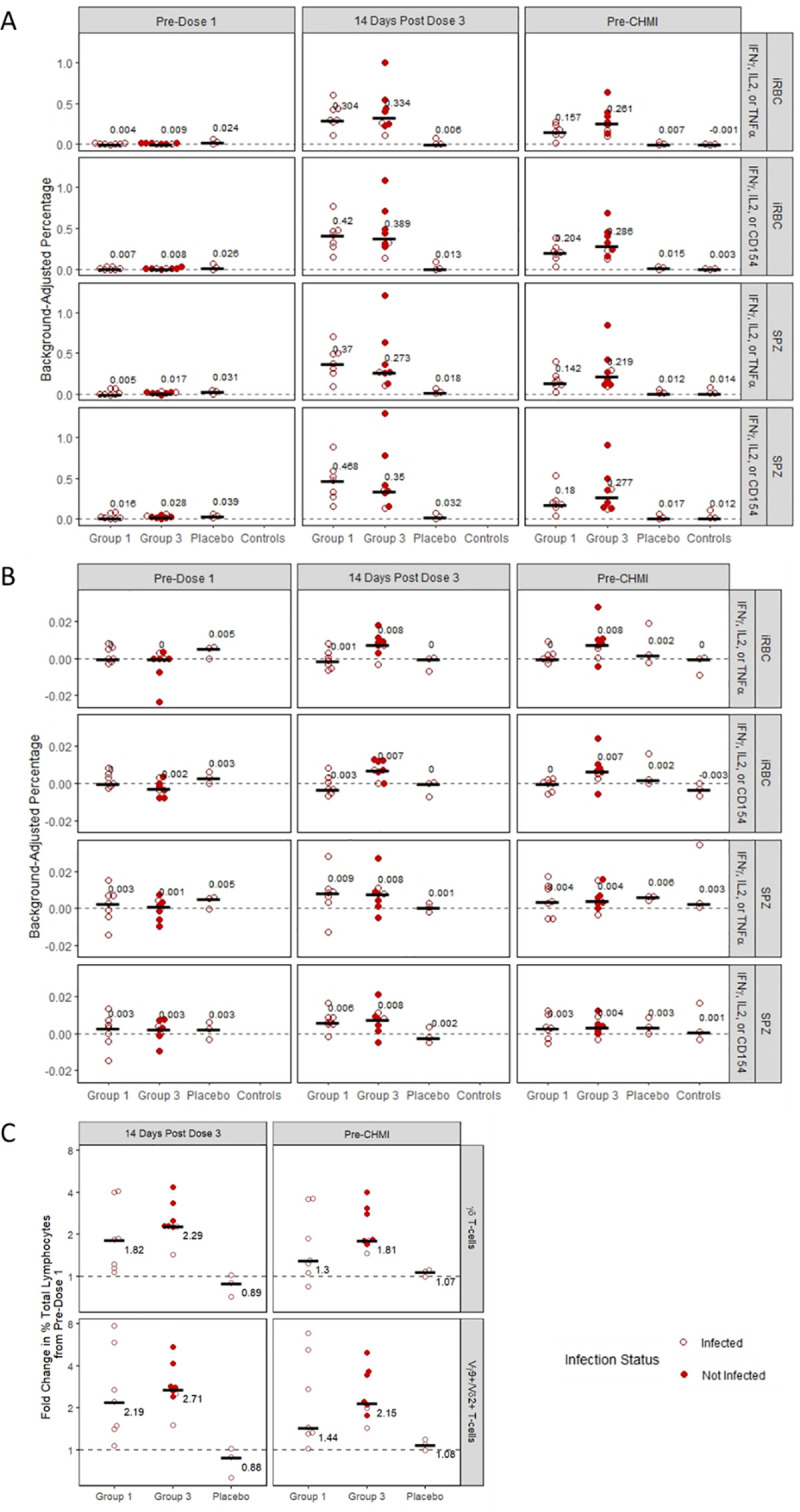
Cellular immune parameters in vaccinated and control participants. (**A**) Background-adjusted percent of CD4 T-cells expressing specific cytokines upon stimulation with malaria antigens by time point among vaccinated subjects in Groups 1 and 3, placebo recipients, and infectivity controls. (**B**) Background-adjusted percent of CD8 T-cells expressing specific cytokines upon stimulation with malaria antigens by time point and treatment group. (**C**) Fold Change in γδ T-cells and in the Vγ9+/Vδ2+ subset from pre-vaccination to 14 days post-vaccination and to pre-CHMI by treatment group. Bars and text show medians in all panels.

For PfSPZ-specific peripheral CD4 responses, trends in background-adjusted percentage responding were similar after *in vitro* stimulation with PfSPZ and Pf-infected red blood cells (iRBC) and between the two cytokine sets (Fig 6A). Responses were highest at the 14 day post-dose 3 time point for vaccinated subjects in Groups 1 and 3 and were similar between those two groups. Although the responses at 14 days post dose 3 for the two subjects in Group 3 who were infected post-CHMI were lower than the mean for their group for all combinations of antigens and cytokine sets, this was not the case for the pre-CHMI time point, and these findings did not appear to correlate with protection when considering that all subjects in Group 1 were infected post-CHMI, despite having comparable results to those in Group 3 who were not infected.

Post-vaccination peripheral CD8 T-cell responses were lower than the CD4 responses in both Groups 1 and 3 (Fig 6B). With iRBC stimulation, for Group 1, there was no increase from pre- to post- vaccination in cytokine-expressing CD8 T-cells while there was a modest response in Group 3. With PfSPZ stimulation, there were modest responses in both groups.

There was a vaccine-induced increase in the frequency of total γδ T cells as a percentage of all lymphocytes, which was largely accounted for by increases in the Vγ9+/Vδ2+ subset, in Groups 1 and 3, and fold changes from pre- to post-vaccination time points were similar in the two groups (Fig 6C). The pre-vaccination proportion of γδ T cells, and of the Vγ9+/Vδ2+ subset, among all lymphocytes were also similar across groups, and between Group 3 vaccinated subjects who were infected and uninfected after CHMI (Table 3).

**Table 3:** Median percent γδ T cells of total lymphocytes and of Vγ9+/Vδ2+ subset of total lymphocytes by time point, Group, and CHMI infection status.

		Subjects given PfSPZ Challenge				Placebo
Cell type	Time point	Group 1 all n = 7^1^	Group 3 all n = 8	Group 3 infected after CHMI n = 2	Group 3 uninfected after CHMI n = 6	Group 1 infected n = 3^1^
**γδ** T-cells (min, max)	Baseline	2.30% (0.83, 6.73)	2.37% (1.34, 5.87)	2.37% (2.04, 2.71)	2.27% (1.34, 5.87)	2.40% (2.01, 2.66)
	14 days post-dose 3	5.64% (2.62, 7.13)	6.00% (2.89, 13.37)	4.52% (2.89, 6.15)	6.06% (3.93, 13.37)	2.04% (1.89, 2.13)
	Pre-CHMI	3.82% (1.94, 8.75)	4.94% (2.95, 9.96)	3.91% (2.95, 4.86)	5.19% (4.80, 9.96)	2.38% (2.15, 2.94)
V**γ**9^+^/V**δ**2^+^ subset (min, max)	Baseline	0.81% (0.49, 6.26)	1.84% (1.03, 5.35)	1.96% (1.70, 2.23)	1.64% (1.03, 5.35)	1.45% (1.41, 1.78)
	14 days post-dose 3	4.80% (0.68, 6.65)	5.42% (2.53, 12.86)	4.06% (2.53, 5.58)	5.42% (3.36, 12.86)	1.47% (0.88, 1.56)
	Pre-CHMI	3.14% (0.49, 8.10)	4.39% (2.42, 9.41)	3.41% (2.42,4.41)	4.39% (4.23, 9.41)	1.66% (1.57,1.77)

^1^All Group 1 vaccinated and placebo subjects were infected after CHMI.

### CQ pharmacokinetics

The plasma concentrations of CQ at each time point demonstrated individual variation but concentrations within an individual across time tended to be relatively stable, and 89 of the 97 (92%) values were at or above the therapeutic target of 33 ng/mL (S7 Fig), which was chosen as approximately four times the IC_50_ of Pf NF54 (8.6 ng/mL). CQ was undetectable in all treated participants pre-CHMI. In the linear model, no evidence was found for an association between CQ level and post-vaccination PPD (S8 Fig). There were no recrudescent parasitemias after the last CQ dose indicating successful clearance in all cases.

## Discussion

In this study, we conducted a dose-escalation trial of PfSPZ-CVac using five- or seven-day interval administration schedules. Three groups were vaccinated, and two of these groups proceeded to CHMI. The first two vaccinated groups (Groups 1 and 2) received three DVI doses seven days apart of 5.12x10^4^ PfSPZ-CVac or 1.024x10^5^ PfSPZ-CVac, respectively, and there was no observed VE in Group 1 following CHMI. Given the lack of VE in Group 1 and the observation that seven-day intervals coincided with blood-stage parasitemia from the preceding vaccination, the study was redesigned mid-trial to shift from a seven-day interval to a five-day interval. Group 3 received three DVI doses of 1.024x10^5^ PfSPZ-CVac five days apart, resulting in 75% VE following CHMI. VE in Group 3 may have increased due to the higher vaccine dose and/or the change to the five-day interval between doses as discussed below.

The expected finding from this trial was that Group 3, which assessed the same three-dose, five-day interval regimen as the prior trial in Germany but with a two-fold increase in the number of PfSPZ per injection, showed a VE of 75%, compared to VE of 63% in the prior trial [2], confirming the efficacy shown in the published data. The unexpected finding was that the same dose of PfSPZ Challenge that demonstrated 63% VE in Germany administered as three doses five days apart had zero VE when administered seven days apart to our Group 1 subjects, suggesting that the lack of VE for the seven-day schedule was due to the difference in the interval between vaccinations, as this was the only variable that was different. Notably, with the seven-day schedule, the second and third vaccinations were given during the period of blood stage parasitemia from the prior vaccination, whereas this was not the case with the five-day schedule. Although it did not induce sterile protection, the seven-day schedule induced partial immunity, as evidenced by the longer prepatent period in vaccinated versus control subjects after CHMI. There are potential limitations to the comparisons made between this study and the study in Germany including an increased dose of PfSPZ-CVac used here in the five-day interval group, possible underlying differences in the malaria-naïve study populations at these two non-endemic sites, and relatively small group sizes.

Group 3 showed a trend toward progressive reductions in the density of transient parasitemia with successive immunizations, particularly from the first to the second, indicating possible effects of innate and/or early adaptive immunity. A similar finding was strongly associated with protection against CHMI in the German study—in the groups immunized every four weeks, all 14 individuals with a ≥100-fold decrease in post-immunization parasitemia between first and third immunization were protected against CHMI, with a similar trend in the five-day interval group. However, Group 1 subjects had similar parasite densities after the first and second vaccinations, indicating little or no impact from the first immunization on development of PfSPZ in the liver following the second immunization.

This absence of sharp declines in parasitemia with the second immunization, coupled with the lack of protection following CHMI, suggests that the induction of liver stage immunity was impaired in Group 1 (seven-day interval) compared to Group 3 (five-day interval). We believe that inoculation of PfSPZ coincident with parasitemia, as evident in Fig 3, is likely related to this negative impact on formation of pre-erythrocytic immunity. This hypothesis is consistent with prior reports of the immunomodulatory influence of erythrocytic parasitemia on the development of functional sporozoite-immune responses to malarial and to non-malarial antigens [27–37]. Wild-type sporozoites can also induce changes in the liver that can innately block entry of subsequent sporozoite doses [38–40]. Fully exploring the realm of possibilities to mechanistically and immunologically explain the differences between Groups 1 and 3 will require additional studies. Perhaps the differences in the five- and seven-day CVac studies will help us better understand how vaccines work in persons with asymptomatic blood stage parasitemia in malaria-endemic regions. A potentially complex mix of innate and adaptive immune factors likely come into play.

Rodent and nonhuman primate models of responses to whole sporozoite vaccines indicate a major role for liver-stage directed CD8 T cells in protection from challenge [41–47]. In humans, liver tissue-specific T cell responses cannot easily be directly evaluated (although preliminary efforts are underway to do so using very small core needle biopsies) [48]. Instead, peripheral T cell responses are assessed, but those responses may not represent responses in the liver [1,45,49]. The role of CD4 T cells following whole sporozoite vaccination is not fully understood but CD4 T cells capable of producing cytokines following *ex vivo* stimulation with blood-stage parasites or sporozoites are elicited by the PfSPZ-CVac approach in humans [1–3]. Liver stage and blood stage infection may also induce humoral responses, such as anti-PfCSP antibody responses, which can inhibit sporozoite infection of hepatocytes [50]. The contribution of anti-PfCSP, or other humoral, responses to protection in humans following PfSPZ-CVac is, however, not clear.

In this study, we assessed cellular and humoral responses to look for associations with PfSPZ dose or protection against CHMI. Nearly all vaccinated subjects seroconverted by producing antibodies against PfCSP by ELISA and PfSPZ by aIFA, and most had serum that inhibited sporozoite invasion *in vitro*. However, despite the marked difference in VE and the evidence for functionally impaired immunity in Group 1, most measures did not reveal substantive differences in those responses between the relatively small number of subjects in Groups 1 (seven-day interval) and 3 (five-day interval).

For T cell responses, there was a similar increase in frequency of PfSPZ- and Pf iRBC- specific memory CD4 T cells expressing any combination of IFN-γ, IL2 or TNFα, or any combination of IFN-γ, IL2, or CD154, following vaccination in both Groups 1 and 3. Among Group 3 subjects there was a suggestion of lower CD4 post-vaccination responses in those infected post-CHMI, but CD4 responses in Group 3 subjects who were protected were generally similar to those in Group 1 subjects, all of whom were infected post-CHMI.

Recent findings from other clinical trials of PfSPZ-CVac [2] or Sanaria’s other vaccine product (PfSPZ Vaccine), composed of irradiated sporozoites [1,51,52], indicate that PfSPZ vaccines induce an increase in the frequency of γδ T cells and that pre- and/or post-vaccination levels of the Vδ2 subset of γδ T cells may be correlated with protection [49,52]. Mouse models suggest that γδ T cells are essential during whole sporozoite vaccination to induce effector CD8 T cells that mediate sterile protection [49]. We also found increases in the frequency of total γδ T cells, which were largely due to increases in the Vγ9Vδ2 subgroup, following vaccination but those responses were similar in Groups 1 and 3. There was also no difference in frequency of total γδ T cells or the Vγ9Vδ2 subgroup at baseline between the two groups, or between protected and non-protected subjects in Group 3. Thus, our immunologic assessments of peripheral markers of cellular and humoral immunity could not explain the differences in protective immunity between the two groups. However, the lack of findings is consistent with the primary effector mechanism residing in liver-resident CD8 T cells, which cannot be accessed from the periphery.

During the vaccination phase, intervals of post-vaccination parasitemia were frequently associated with solicited systemic AEs and for some participants these symptoms interfered with their usual activities (Grade 3). Of the 20 subjects who received PfSPZ Challenge by DVI, during the 7–10 day post-vaccination interval, all reported at least one solicited systemic AE, five (25%) reported at least one Grade 3 solicited systemic AE, and three (15%) were discontinued from the vaccination series due to an AE. In contrast, during that same interval, solicited systemic AEs were infrequently reported in Group 1 placebo recipients. After vaccine administration but prior to onset of parasitemia, solicited systemic AEs were also infrequently reported in the vaccinated groups, indicating that the injected PfSPZ and the developing liver stage parasites were not reactogenic. Unless they can be reduced, AEs resulting from the transient parasitemia allowed by CQ prophylaxis could limit broader adaption of the PfSPZ-CVac (CQ) approach in individuals lacking prior exposure to malaria.

The safety information from subjects in the two condensed schedule groups evaluated in the Germany PfSPZ-CVac trial has not yet been published but the subjects given three PfSPZ Challenge injections four weeks apart were reported to have an AE profile like the corresponding placebo group [2], which differed from what was observed for the accelerated schedules tested here. In contrast, the vaccine-associated AE profiles reported by three earlier studies of administration of infectious sporozoites by mosquito bites, administered in three biting sessions four weeks apart, to subjects taking concomitant CQ, are similar to our findings. One of the three studies reported that all 10 subjects (100%) had at least one malaria-like symptom associated with parasitemia and three (30%) had severe AEs, with the highest frequency of AEs after the first vaccination, when the highest parasite densities occurred [3]. A second study reported that all 15 subjects had at least one systemic AE associated with parasitemia after the first vaccination and four (27%) experienced a grade 3 AE [10]. Lastly, the third study, in which subjects took either CQ or mefloquine, found that 13 of 14 (93%) subjects had at least one malaria-like AE following the first vaccination with 2 (14%) reporting a severe AE [11].

Use of a sensitive qRT-PCR assay during the CHMI phase allows earlier detection of blood stage parasitemia and more rapid initiation of treatment compared with the traditional use of thick blood smears [20,53]. When we found no evidence of false positive qRT-PCR results in the Group 1 CHMI (all subjects with an initial positive result at the estimated ≥20 parasites/mL threshold subsequently reached the estimated ≥250 threshold), we changed the criterion for positivity to the ≥20 threshold for Group 3, allowing earlier treatment and thereby achieving a substantial reduction in the number and severity of malaria-related adverse events: no subject had a grade 3 solicited systemic AE, and none had a parasite density >500 parasites/mL.

The PfSPZ-CVac approach has been shown to induce sterilizing immunity at a dose of 5.12x10^4^ PfSPZ administered as three DVIs 28 days apart [2], indicating the potential of this concept. In our assessment of condensed schedules, we found that timing of administration of the live, infectious PfSPZ Challenge vaccine was critical, and that administration of the dose of 5.12x10^4^ PfSPZ every seven days was ineffective, while administration of double that dose every five days gave a VE of 75%. This contrast suggests that there is significant interference with a required immune mechanism when parasite liver and blood stages are simultaneously present. Our evaluation of humoral responses to blood stage antigens and functional responses, as well as ICS responses, did not identify differences between the two groups suggesting a mechanism for the lack of VE in Group 1. Further evaluations of study specimens, including serum cytokine profiling, are planned but, as has been previously suggested [54,55], it may be difficult to identify immune response signatures that predict VE unless responses in the liver can be assessed, which suggests a role for animal models to further explore this phenomenon.

## Supporting information

S1 CONSORT Checklist (DOC)

S1 Fig Antibodies to PfCSP (net OD 1.0) pre-immunization, two weeks after the third dose of PfSPZ-CVac, prior to CHMI and 28 days post CHMI. (TIF)

S2 Fig Antibodies to PfSPZ (net aIFA200K) pre-immunization, two weeks after the third dose of PfSPZ-CVac, prior to CHMI and 28 days post CHMI. (TIF)

S3 Fig Reciprocal serum dilution for 80% inhibition of PfSPZ invasion of hepatocytes (HC-04 cells), pre-immunization, two weeks after third dose of PfSPZ-CVac, pre-CHMI and 28 days post CHMI. (TIF)

S4 Fig Antibodies to PfCSP by ELISA (net OD 1.0) two weeks after the third vaccine dose. (TIF)

S5 Fig Antibodies to PfSPZ (net aIFA200K) two weeks after third vaccine dose. (TIF)

S6 Fig Net reciprocal serum dilution for 80% inhibition of PfSPZ invasion of hepatocytes (HC-04 cells) two weeks after the third vaccine dose. (TIF)

S7 Fig Plasma chloroquine (CQ) concentrations at four time points (two days after each of four chloroquine doses) during the vaccination phase. (TIF)

S8 Fig Individual line plot of square root peak parasite density by CQ level, group, and vaccination number. (TIF)

S1 Table Subject demographic characteristics. (DOCX)

S2 Table Overall number and percentage of subjects experiencing systemic or local AEs after any PfSPZ CVac immunization or placebo dose by study group. (DOCX)

S3 Table Peak parasite densities (PPD), solicited systemic adverse events (AEs), and vaccine-related unsolicited AEs occurring 7–10 days after each PfSPZ Challenge vaccine-phase injection by study group and subject. (DOCX)

S4 Table Post-CHMI peak parasite density (PPD) (parasites/mL), maximum grade of each reported solicited systemic adverse events (AE), and temporal patterns of parasitemia for Groups 1 and 3 by infected participant. (DOCX)

S5 Table Immunological data for all volunteers in Seattle PfSPZ CVac trial, as measured by PfCSP ELISA, aIFA, and ISI assays. (DOCX)

S1 Text Additional information about randomization and blinding. (DOCX)
