# Supplementary figures and images for: PfSPZ-CVac efficacy against malaria increases from 0% to 75% when administered in the absence of erythrocyte stage parasitemia: A randomized, placebo-controlled trial with controlled human malaria infection
Source: PLoS Pathog. 2021 May 28;17(5):e1009594. doi: 10.1371/journal.ppat.1009594 (PMC8191919; doi:10.1371/journal.ppat.1009594)

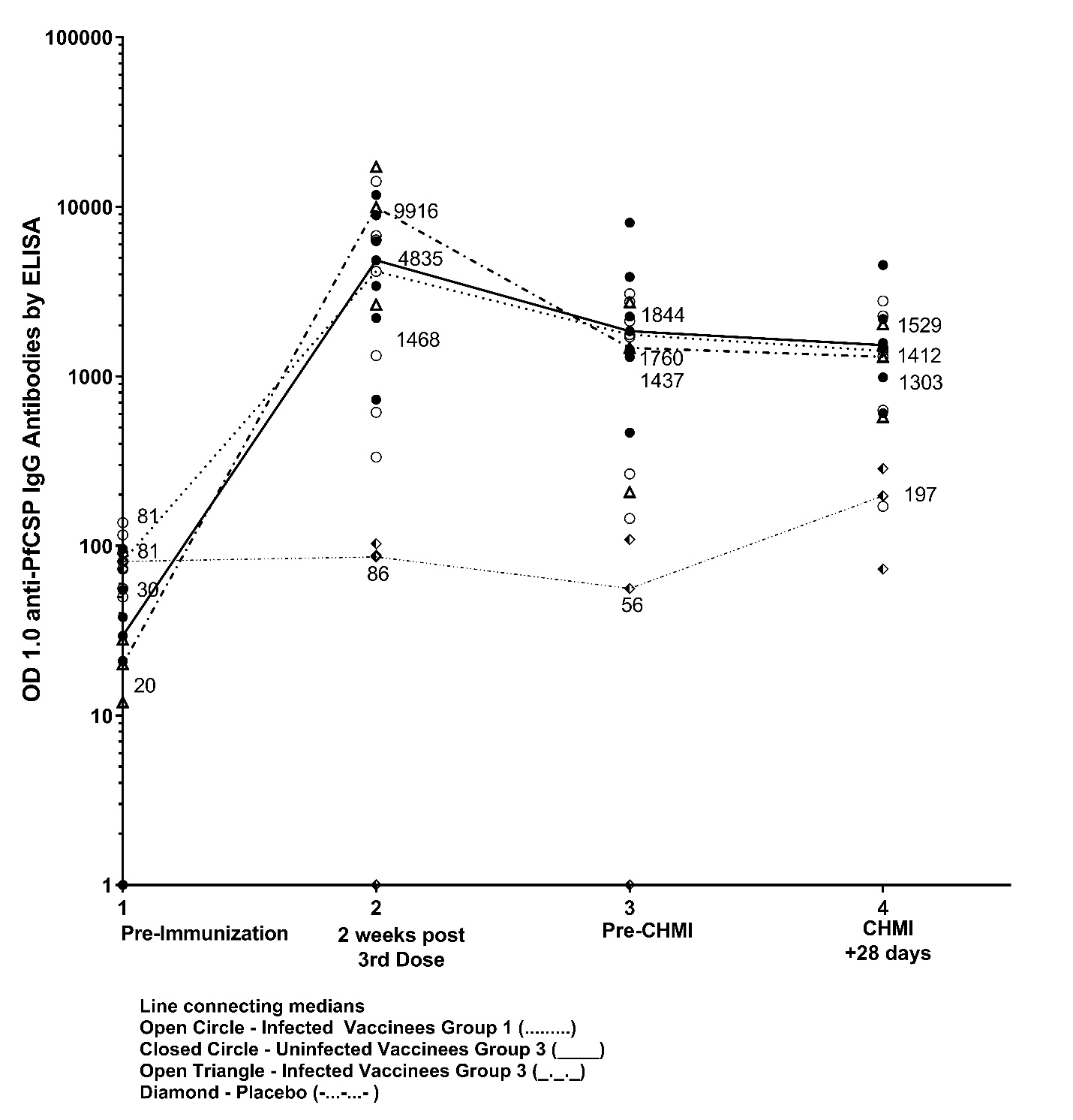

Supplement: S1 Fig — (TIF) [file ppat.1009594.s002.tif]

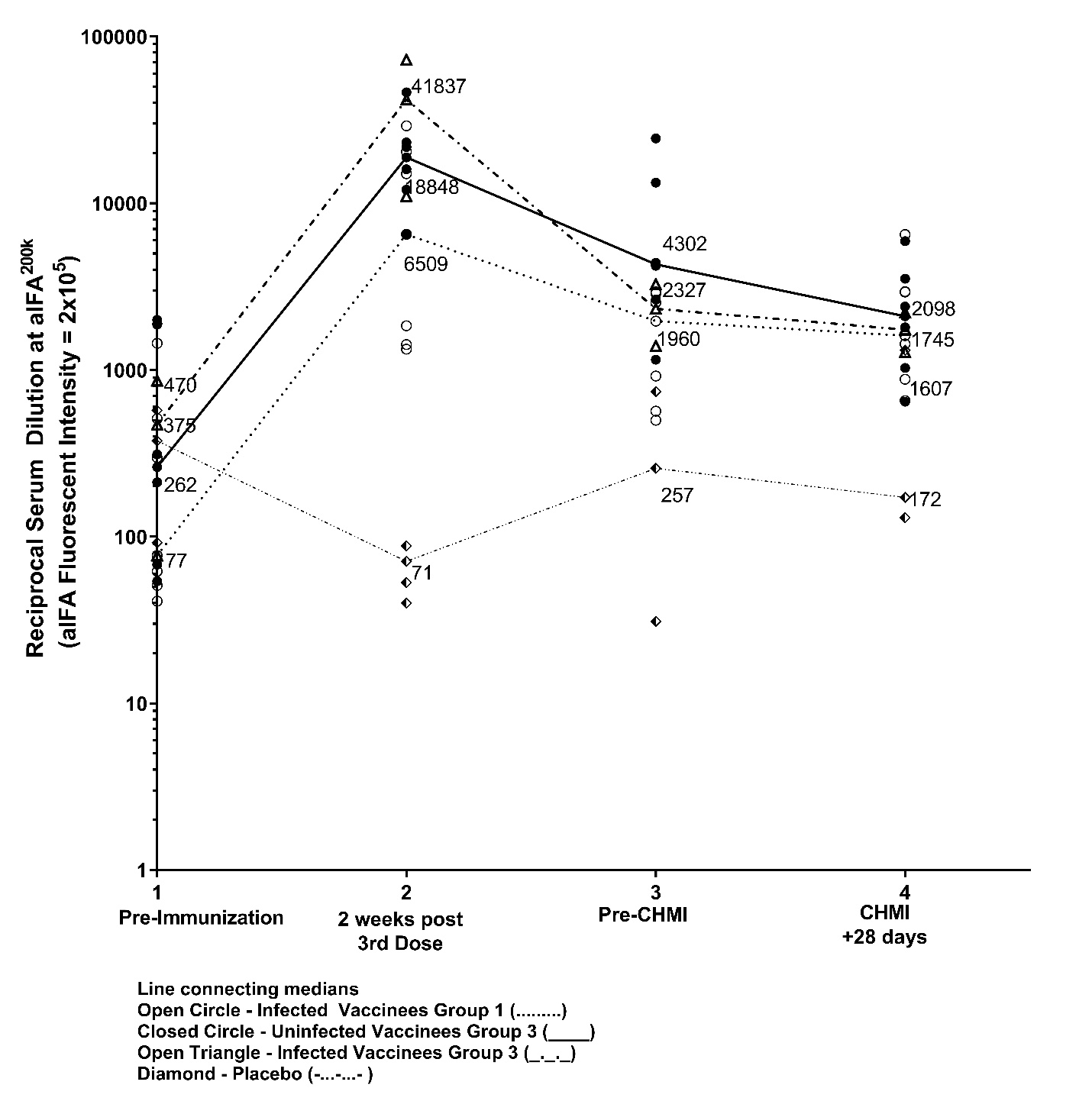

Supplement: S2 Fig — (TIF) [file ppat.1009594.s003.tif]

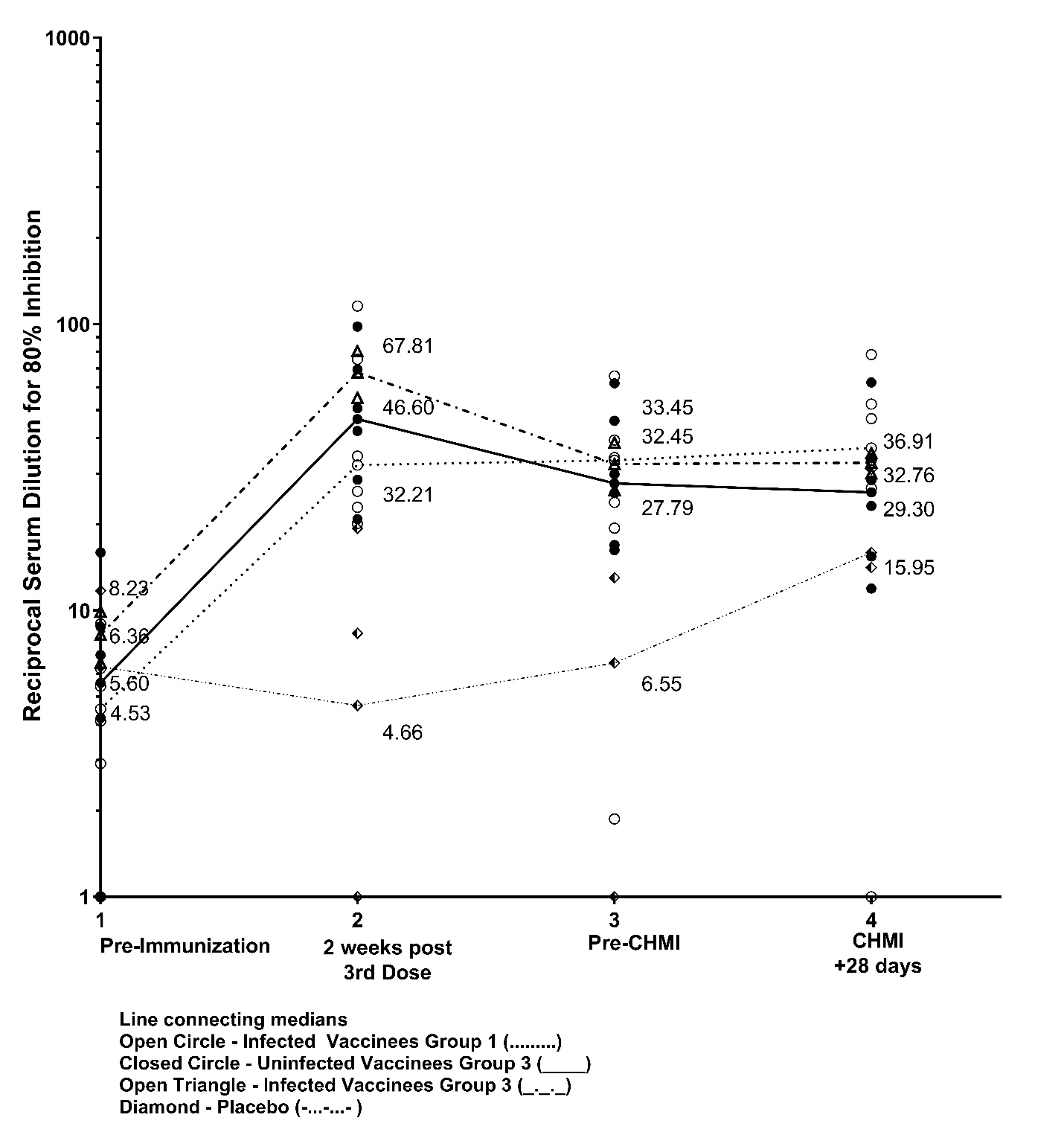

Supplement: S3 Fig — (TIF) [file ppat.1009594.s004.tif]

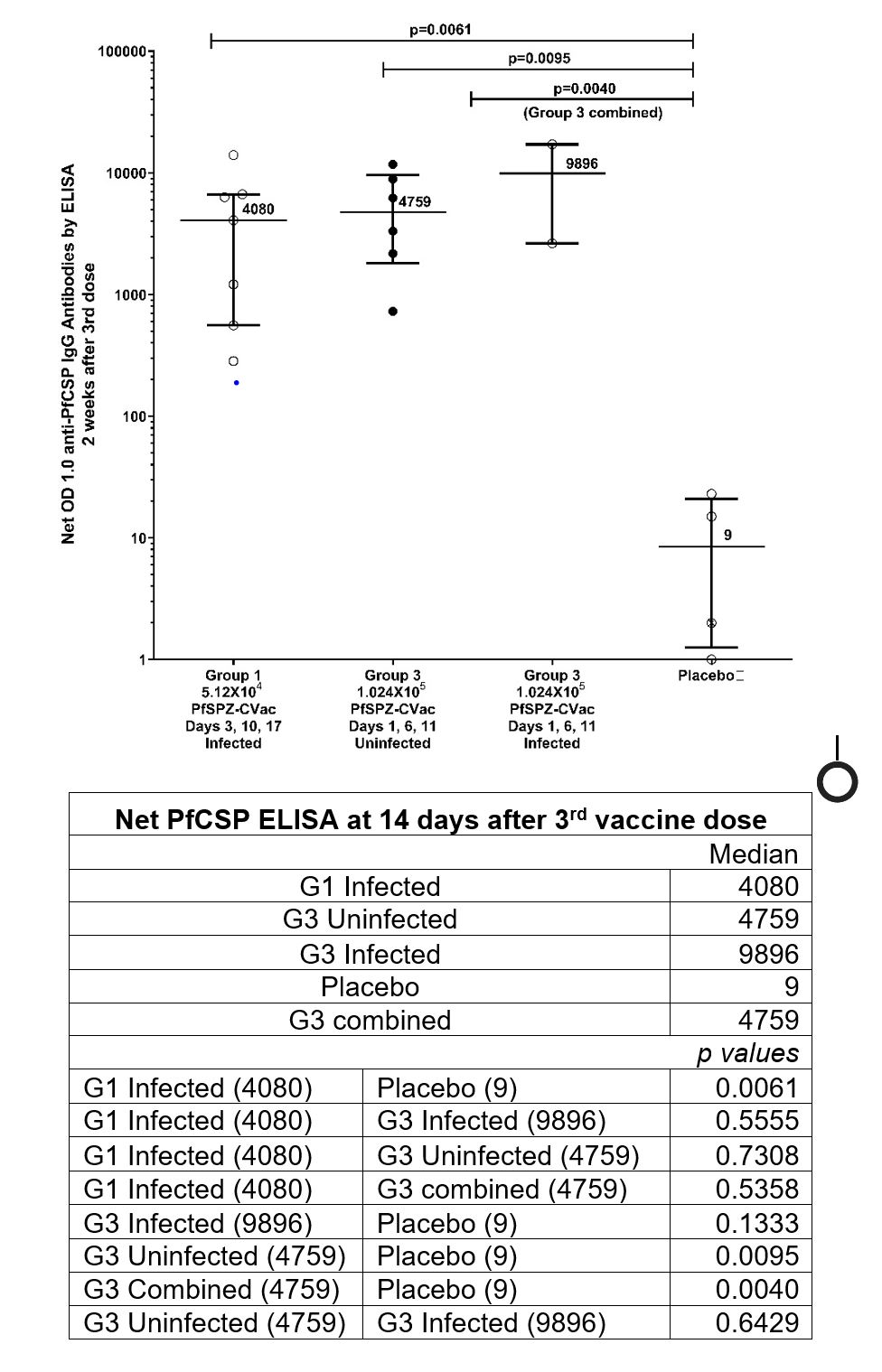

Supplement: S4 Fig — (TIF) [file ppat.1009594.s005.tif]

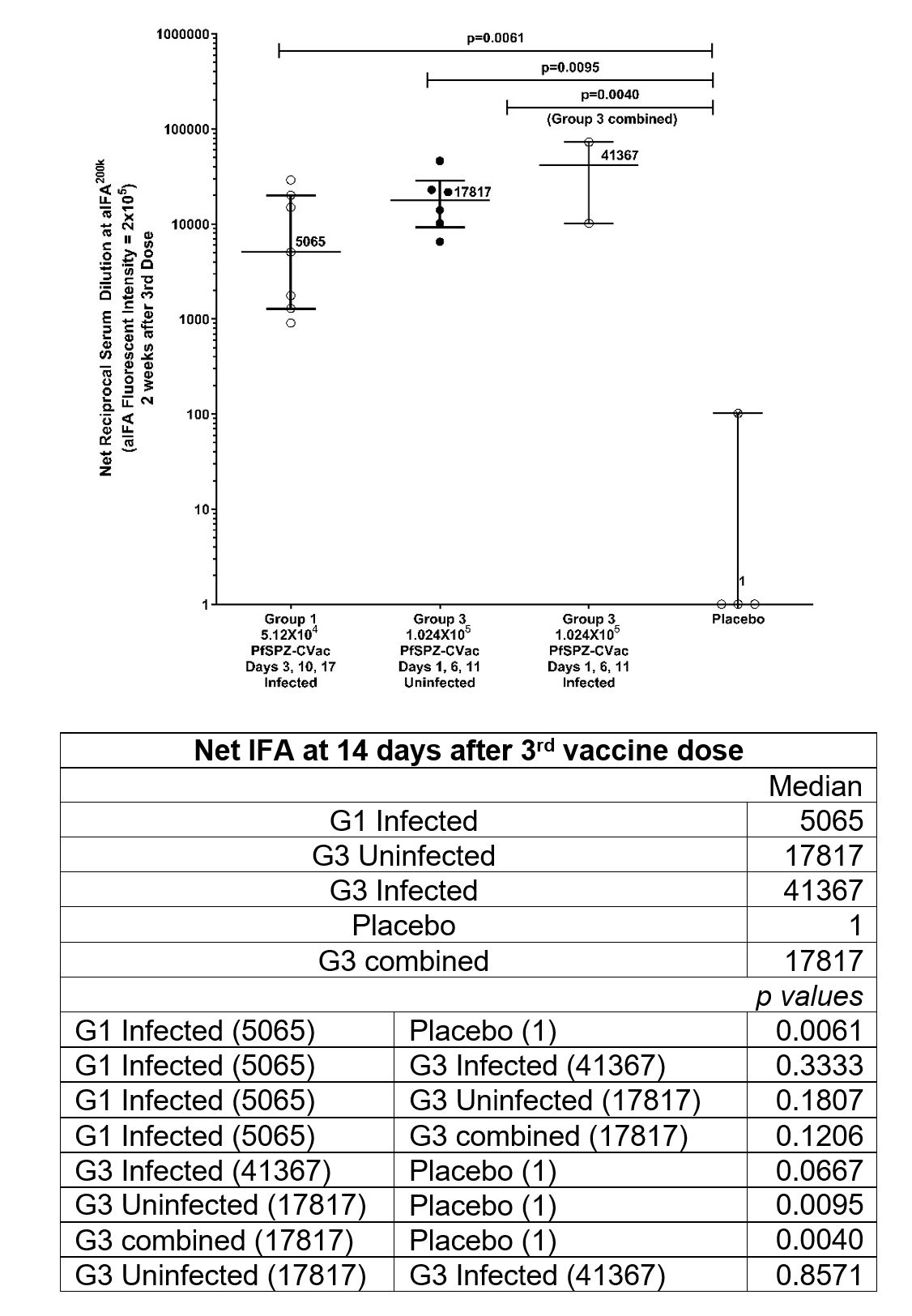

Supplement: S5 Fig — (TIF) [file ppat.1009594.s006.tif]

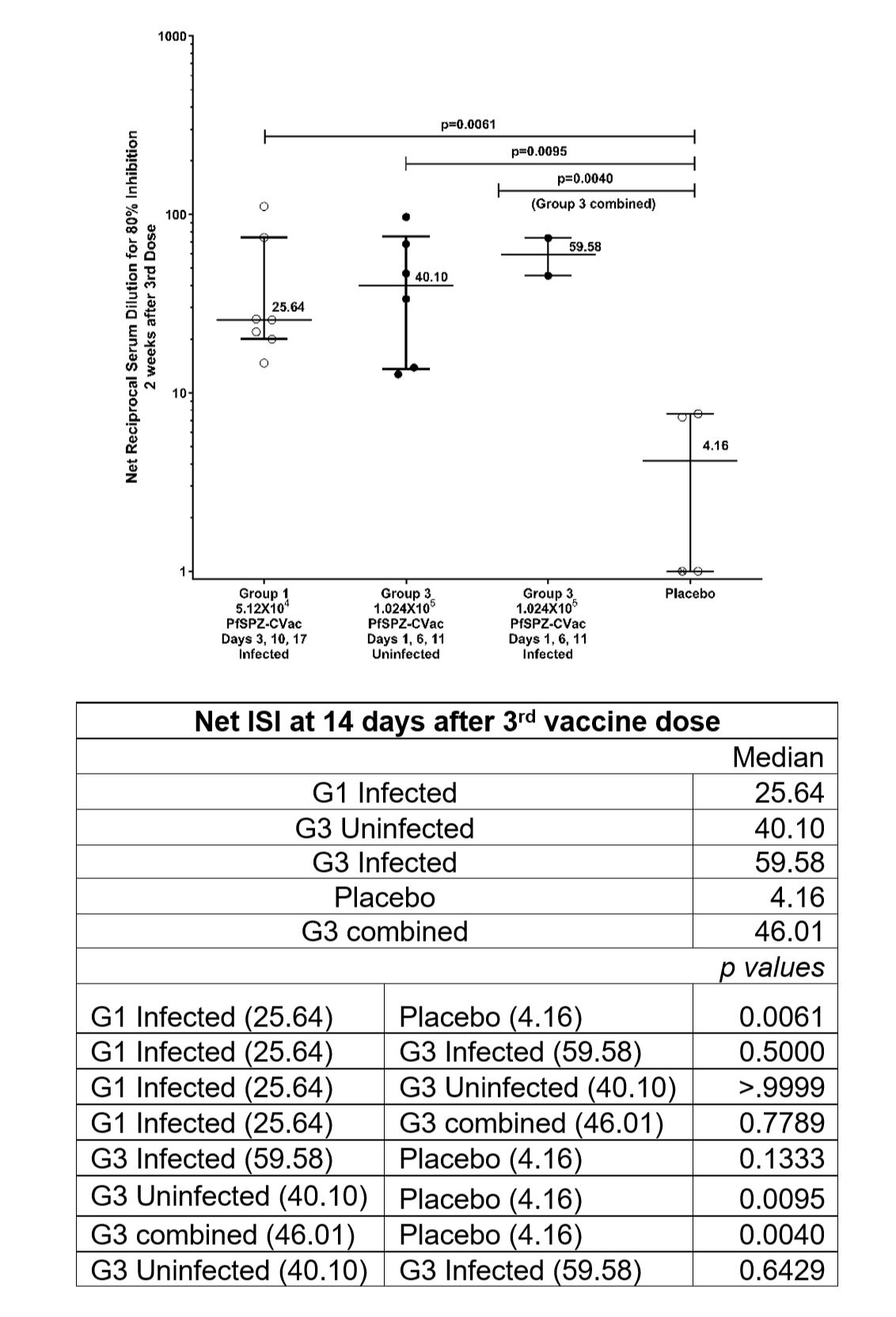

Supplement: S6 Fig — (TIF) [file ppat.1009594.s007.tif]

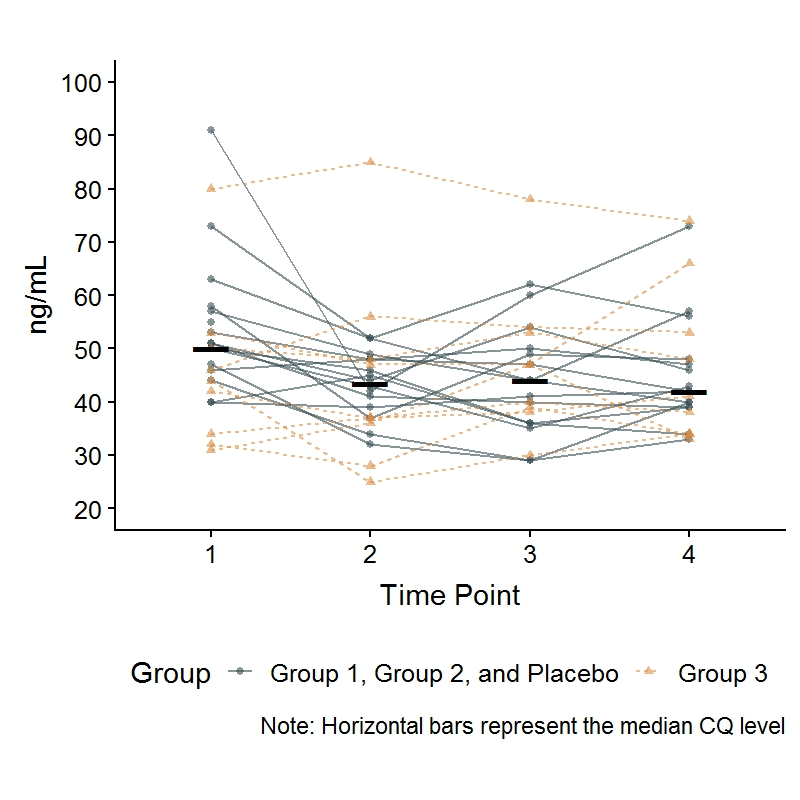

Supplement: S7 Fig — (TIF) [file ppat.1009594.s008.tif]

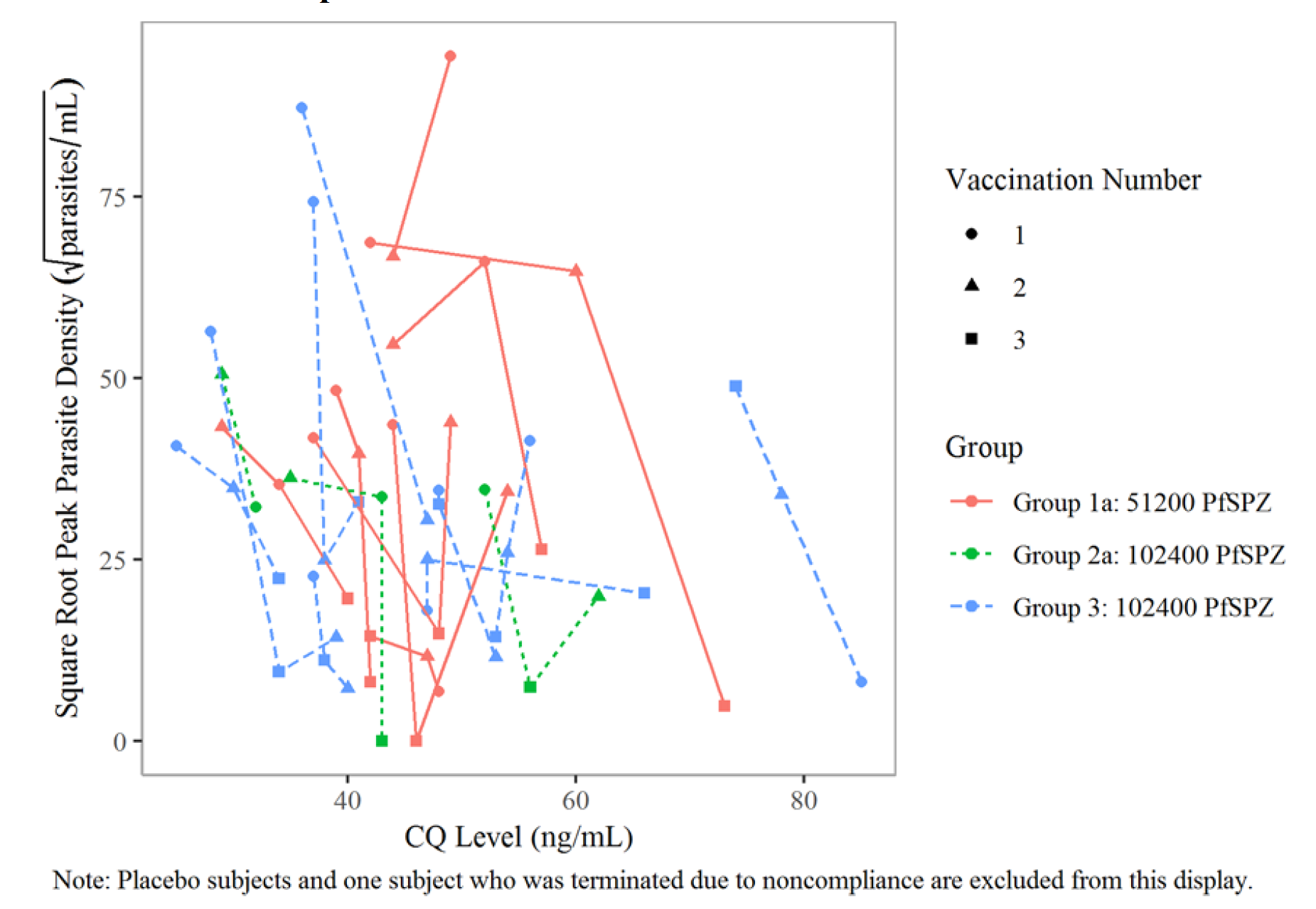

Supplement: S8 Fig — (TIF) [file ppat.1009594.s009.tif]
