# Supplementary material for: PfSPZ-CVac efficacy against malaria increases from 0% to 75% when administered in the absence of erythrocyte stage parasitemia: A randomized, placebo-controlled trial with controlled human malaria infection
Source: PLoS Pathog. 2021 May 28;17(5):e1009594. doi: 10.1371/journal.ppat.1009594 (PMC8191919; doi:10.1371/journal.ppat.1009594)
Supplement: S1 Table — (DOCX) [file ppat.1009594.s010.docx]

**Table S1. Subject demographic characteristics**

| **Category** | **Group 1** | **Group 2** | **Group 1/2** | **Group 3** | |
| --- | --- | --- | --- | --- | --- |
| **Treatment** | **Vaccinees** | **Vaccinees** | **Placebos** | **Vaccinees** | **Infectivity Controls** |
| **N** | 9 | 3 | 4 | 9 | 3 |
| **% Male** | 44.4% | 33.3% | 75.0% | 55.6% | 66.7% |
| **% White** | 88.9% | 100.0% | 75.0% | 100.0% | 100.0% |
| **% Not Hispanic or Latino** | 77.8% | 100.0% | 100.0% | 100.0% | 100.0% |
| **Mean age (range)** | 29.6 (20-34) | 32.7 (28-38) | 29.0 (24-35) | 27.3 (19-33) | 28.0 (25-33) |
