# Supplementary material for: PfSPZ-CVac efficacy against malaria increases from 0% to 75% when administered in the absence of erythrocyte stage parasitemia: A randomized, placebo-controlled trial with controlled human malaria infection
Source: PLoS Pathog. 2021 May 28;17(5):e1009594. doi: 10.1371/journal.ppat.1009594 (PMC8191919; doi:10.1371/journal.ppat.1009594)
Supplement: S2 Table — (DOCX) [file ppat.1009594.s011.docx]

**Table S2. Overall number and percentage of subjects experiencing systemic or local AEs after any PfSPZ CVac immunization or placebo dose by study group**

| **Symptom** | **Group 1 Vaccinees (n=9)** | | | **Group 2 Vaccinees (n=3)** | | | **Group 3 Vaccinees (n=9)** | | | **Group 1/2 Placebo (n=4)** | | |
| --- | --- | --- | --- | --- | --- | --- | --- | --- | --- | --- | --- | --- |
|  | n | % | 95%CI | n | % | 95%CI | n | % | 95%CI | n | % | 95%CI |
| **Any symptom** | 8 | 88.9 | 56.50, 98.01 | 3 | 100.0 | 43.85, 100.00 | 9 | 100.0 | 70.09, 100.00 | 4 | 100.0 | 51.05, 100.00 |
| **Any systemic symptoms** | 8 | 88.9 | 56.50, 98.01 | 3 | 100.0 | 43.85, 100.00 | 9 | 100.0 | 70.09, 100.00 | 3 | 75.0 | 30.06, 95.44 |
| - Arthralgia/joint pain | 5 | 55.6 | 26.67, 81.12 | 2 | 66.7 | 20.77, 93.85 | 6 | 66.7 | 35.42, 87.94 | 0 | 0.0 | 0.00, 48.99 |
| - Chills | 6 | 66.7 | 35.42, 87.94 | 3 | 100.0 | 43.85, 100.00 | 6 | 66.7 | 35.42, 87.94 | 0 | 0.0 | 0.00, 48.99 |
| - Headache | 8 | 88.9 | 56.50, 98.01 | 3 | 100.0 | 43.85, 100.00 | 9 | 100.0 | 70.09, 100.00 | 1 | 25.0 | 4.56, 69.94 |
| - Malaise | 8 | 88.9 | 56.50, 98.01 | 3 | 100.0 | 43.85, 100.00 | 8 | 88.9 | 56.50, 98.01 | 1 | 25.0 | 4.56, 69.94 |
| - Myalgia/body aches | 8 | 88.9 | 56.50, 98.01 | 2 | 66.7 | 20.77, 93.85 | 8 | 88.9 | 56.50, 98.01 | 0 | 0.0 | 0.00, 48.99 |
| - Nausea | 2 | 22.2 | 6.32, 54.74 | 1 | 33.3 | 6.15, 79.23 | 4 | 44.4 | 18.88, 73.33 | 1 | 25.0 | 4.56, 69.94 |
| - Fever | 3 | 33.3 | 12.06, 64.58 | 2 | 66.7 | 20.77, 93.85 | 1 | 11.1 | 1.99, 43.50 | 0 | 0.0 | 0.00, 48.99 |
| - Vomiting | 0 | 0.0 | 0.00, 29.91 | 0 | 0.0 | 0.00, 56.15 | 0 | 0.0 | 0.00, 29.91 | 0 | 0.0 | 0.00, 48.99 |
| **Any local symptom** | 7 | 77.8 | 45.26, 93.68 | 1 | 33.3 | 6.15, 79.23 | 5 | 55.6 | 26.67, 81.12 | 2 | 50.0 | 15.00, 85.00 |
| - Ecchymosis/bruising measurement | 1 | 11.1 | 1.99, 43.50 | 0 | 0.0 | 0.00, 56.15 | 1 | 11.1 | 1.99, 43.50 | 0 | 0.0 | 0.00, 48.99 |
| - Ecchymosis/bruising severity | 5 | 55.6 | 26.67, 81.12 | 1 | 33.3 | 6.15, 79.23 | 4 | 44.4 | 18.88, 73.33 | 0 | 0.0 | 0.00, 48.99 |
| - Erythema/redness measurement | 0 | 0.0 | 0.00, 29.91 | 0 | 0.0 | 0.00, 56.15 | 0 | 0.0 | 0.00, 29.91 | 0 | 0.0 | 0.00, 48.99 |
| - Erythema/redness severity | 5 | 55.6 | 26.67, 81.12 | 1 | 33.3 | 6.15, 79.23 | 5 | 55.6 | 26.67, 81.12 | 2 | 50.0 | 15.00, 85.00 |
| - Induration/swelling measurement | 0 | 0.0 | 0.00, 29.21 | 0 | 0.0 | 0.00, 56.15 | 0 | 0.0 | 0.00, 29.91 | 0 | 0.0 | 0.00, 48.99 |
| - Induration/swelling severity | 2 | 22.2 | 6.32, 54.74 | 1 | 33.3 | 6.15, 79.23 | 2 | 22.2 | 6.32, 54.74 | 1 | 25.0 | 4.56, 69.94 |
| - Pain at injection site | 0 | 0.0 | 0.00, 29.91 | 1 | 33.3 | 6.15, 79.23 | 0 | 0.0 | 0.00, 29.91 | 0 | 0.0 | 0.00, 48.99 |
| - Tenderness at injection site | 4 | 44.4 | 18.88, 73.33 | 1 | 33.3 | 6.15, 79.23 | 3 | 33.3 | 12.06, 64.58 | 1 | 25.0 | 4.56, 69.94 |

N = number of subjects in the safety population who received at least one dose.

Wilson score interval used for confidence interval of proportion.
