# Supplementary material for: PfSPZ-CVac efficacy against malaria increases from 0% to 75% when administered in the absence of erythrocyte stage parasitemia: A randomized, placebo-controlled trial with controlled human malaria infection
Source: PLoS Pathog. 2021 May 28;17(5):e1009594. doi: 10.1371/journal.ppat.1009594 (PMC8191919; doi:10.1371/journal.ppat.1009594)
Supplement: S3 Table — (DOCX) [file ppat.1009594.s012.docx]

**Table S3. Peak parasite densities (PPD), solicited systemic adverse events (AEs), and vaccine-related unsolicited AEs occurring 7-10 days after each PfSPZ Challenge vaccine-phase injection by study group and subject**

| Group | Subject | 7-10 days after  1^st^ injection | | 7-10 days after  2^nd^ injection | | 7-10 days after 3^rd^ injection | | Unsolicited AEs judged related to PfSPZ-Challenge in the 7-10 day interval after any dose |
| --- | --- | --- | --- | --- | --- | --- | --- | --- |
|  |  | **PPD** | **AE** | **PPD** | **AE** | **PPD** | **AE** |  |
| **Group 1 (5.12 x 10^4^ PfSPZ-CVac administered DVI on Days 3, 10, 17)*** | 1* | 8929 | 38.1 M My A C H | 4467 | My A | Discontinued | | Diffuse paresthesias |
|  | 2 | 4373 | H | 2985 | M My A C H | 696 |  | None |
|  | 3 | 46 | M | 135 |  | 209 | M My C H | Tachycardia |
|  | 4 | 1252 |  | 1873 | 38.1 M My A C H | 385 | M My A H | Momentary lightheadedness |
|  | 5 | 2332 | M My A | 1570 | My N M H | 66 |  | None |
|  | 6 | 4727 | My H | 4192 | My M H | 23 | H | Testicular pain, tachycardia |
|  | 7 | 1900 | H | 1183 | My C M H | ND |  | None |
|  | 8 | 1745 |  | 1927 | 39.0 M My C H A | 219 | 39.1 M A C H | Lightheadedness, hand paresthesias, night sweats, anorexia, tachycardia, feeling of “heavy legs” ^NOTE^ ^1^ |
| **Group 2 (1.024 x 10^5^ PfSPZ-CVac administered DVI on Days 3, 10, 17)** | 9 | 1042 | 38.7 M C N H | 2556 | M C N | Discontinued | | Feeling of chest constriction and inability to take a deep breath, fluttering feeling in upper sternal area, dyspnea on exertion, feeling of tracheal constriction with wheezing ^NOTE^ ^2^ |
|  | 10 | 1200 | C M My H | 398 | A | 55 | M | None |
|  | 11 | 1134 | 38.2 C M My A H | 1315 |  | ND |  | Bilateral hand paresthesias |
| **Group 3 (1.024 x 10^5^ PfSPZ-CVac administered DVI on Days 1, 6, 11)** | 12** | 3184 | M My A C H | 203 | H | 91 |  | None |
|  | 13 | 5528 | H | 622 | My C N H | 1087 | H | None |
|  | 14 | 7621 | 39.4 C M My A H | 925 |  | Discontinued | | Worsening tinnitus, night sweats, occipital tenderness, transient upper sternal chest tightness or pain, neck muscle tightness ^NOTE^ ^3^ |
|  | 15 | 1652 | My M A | 1215 | H | 501 |  | None |
|  | 16** | 322 | M My C | 626 | My H | 415 |  | None |
|  | 17** | 515 | My A H | 53 | H | 124 | H | Night sweats |
|  | 18** | 66 | M A N H | 1157 | M My A C N H | 2392 | H | Transient lightheadedness, feeling hot, multiple episodes of second of a feeling of chest tightness associated with a feeling of needing to take a deep breath. ECG was normal and troponin and CK-MB were not elevated. |
|  | 19** | 1711 | My H | 671 |  | 205 |  | None |
|  | 20** | 1190 | M N H | 133 | M | 1064 | M H | None |

ND = not detected; *One Group 1 vaccine recipient was discontinued for noncompliance, was treated with atovaquone and proguanil after the first injection, and is not shown in the table; **Protected against CHMI

PPDs categorized as: ≥detectable limit and <2000, ≥2000 and <4000, and ≥4000 parasites/mL. Solicited systemic AEs include: Elevated oral temperature (highest value °C reported), Malaise (M), Myalgia (My), Chills (C), Arthralgia (A), Nausea (N), Headache (H), and Vomiting (V). Severity grades are noted by color: Grade 1, Grade 2, Grade 3**.** Temperatures noted only if ≥38.0°C (lower limit of graded fever; Grade 1: 38.0°C - 38.4°C, Grade 2: 38.5°C - 38.9°C, Grade 3 >38.9°C).

**NOTE 1:** On the day of onset of parasitemia due to the second vaccination and prior to the administration of the third vaccination on that day, the subject noted the onset of feeling cold and feeling as if his legs were “heavy”. Later in the day, in addition to the AEs noted, he reported lightheadedness**,** hand paresthesias, anorexia, tachycardia, and again the feeling of “heavy legs.” The next day he noted anorexia and night sweats, and clinical laboratory evaluations for safety were notable only for a grade 1 elevated total bilirubin of 1.4 mg/dL.

**NOTE 2:** On the day of onset of parasitemia due to the first vaccination, the subject noted symptoms of a sensation of constriction in the upper chest, an inability to take a deep breath, and a “fluttering” feeling in the upper sternal area. The symptoms persisted intermittently the next day and he also noted dyspnea on exertion and 30 minutes of a sensation of tracheal constriction associated with expiratory wheezing. He had received the second vaccination prior to onset of symptoms but was discontinued from the third vaccination. With the onset of parasitemia due to the second vaccination, he again noted a sensation of constriction of the upper airway, without wheezing, as well as dyspnea on exertion. He also again noted the feeling of “fluttering” in the upper sternal area, which was momentary but recurrent and continued intermittently for the next five days. He was evaluated by cardiology and pulmonary specialists and had normal troponin and CK-MB values from specimens obtained on the day after the initial onset, multiple normal ECGs, a normal chest radiograph, as well as an unremarkable 48-hour Holter monitor recording and normal pulmonary function testing.

**NOTE 3:** On the day after onset of parasitemia following the first vaccination, in addition to the AEs, the subject noted worsening of his chronic bilateral tinnitus, which continued for three days. The next day he also noted night sweats, which persisted for three days, bilateral occipital tenderness, which persisted for two days, as well as transient chest “tightness” or “pain” in the upper sternal area. The next day he noted neck muscle tightness, which persisted for three days. No abnormalities were noted on physical examination, an ECG was normal, and CK-MB and troponin levels were not elevated. He had received the second vaccination prior to onset of the unsolicited symptoms but was discontinued from the third vaccination. The night sweats and neck muscle tightness persisted after the resolution of parasitemia following the first vaccination and through the first two days of parasitemia due to the second vaccination.
