# Supplementary material for: PfSPZ-CVac efficacy against malaria increases from 0% to 75% when administered in the absence of erythrocyte stage parasitemia: A randomized, placebo-controlled trial with controlled human malaria infection
Source: PLoS Pathog. 2021 May 28;17(5):e1009594. doi: 10.1371/journal.ppat.1009594 (PMC8191919; doi:10.1371/journal.ppat.1009594)
Supplement: S4 Table — (DOCX) [file ppat.1009594.s013.docx]

**Table S4. Post-CHMI peak parasite density (PPD) (parasites/mL), maximum grade of each reported solicited systemic adverse events (AE), and temporal patterns of parasitemia for Groups 1 and 3 by infected participant**

| **Group** | **ID** | **PPD (est. parasites/mL)** | **AEs associated with parasitemia** | **First positive qRT-PCR** | **Treatment threshold met** | **Last positive qRT-PCR** |
| --- | --- | --- | --- | --- | --- | --- |
|  |  |  |  | Day post-CHMI | | |
| **Group 1 (5.12 x 10^4^ PfSPZ-CVac administered DVI on Days 3, 10, 17) and Placebo Controls as indicated** | 2 | 3691 | 38.2 C M My A H N | 10 | 11 | 14 |
|  | 3 | 11434 | 38.6 M My C H | 11 | 12 | 14 |
|  | 4 | 12099 | 38.4 M My A C N | 8 | 11 | 15 |
|  | 5 | 27660 | 39.0 M My C N H A V | 11 | 13 | 16 |
|  | 6 | 9250 | My C A N H | 11 | 13 | 19 |
|  | 7 | 33005 | 38.4 M C H My A N | 13 | 15 | 21 |
|  | 8 | 999 | 39.7 M My C H A N | 10 | 11 | 14 |
|  | 21 (placebo) | 12359 | M N | 8 | 11 | 15 |
|  | 22 (placebo) | 25054 | 38.9 My M C A H N | 8 | 11 | 16 |
|  | 23 (placebo) | 12272 | My H N | 7 | 10 | 13 |
| **Group 3 (1.024 x 10^5^ PfSPZ-CVac administered DVI on Days 1, 6, 11) and Infectivity Controls as indicated** | 13 | 251 |  | 9 | 9 | 10 |
|  | 15 | 242 | M H | 11 | 11 | 13 |
|  | 24 (inf. control) | 161 | M H N | 7 | 7 | 9 |
|  | 25 (inf. control) | 492 | M N | 7 | 7 | 10 |
|  | 26 (inf. control) | 129 | M C H | 7 | 7 | 8 |

SSAEs include: Elevated oral temperature (°C), Malaise (M), Myalgia (My), Chills (C), Arthralgia (A), Nausea (N), Headache (H), and Vomiting (V)

Severity grades are noted by color: Grade 1, Grade 2, Grade 3

Temperature values are noted only if ≥38.0°C (lower limit of graded fever). Fever grading: Grade 1: 38.0°C – 38.4°C, Grade 2: 38.5°C – 38.9°C, Grade 3 >38.9°C

For this presentation of data, PPDs were categorized as: ≥detectable limit and <2000, ≥2000 and <10000, and ≥10000

For Group 1, the treatment definition for CHMI was a positive qRT-PCR with a parasite density of ≥250 parasites/mL on a blood sample obtained within 28 days following CHMI (index sample) that was confirmed by a positive qRT-PCR assay (of any parasite density) from another blood sample that was collected at least 6 hours and no more than 60 hours before or after the index sample. For Group 3, the treatment definition for CHMI was one positive qRT-PCR with a parasite density of ≥20 parasites/mL within 28 days following CHMI.
