# Supplementary material for: PfSPZ-CVac efficacy against malaria increases from 0% to 75% when administered in the absence of erythrocyte stage parasitemia: A randomized, placebo-controlled trial with controlled human malaria infection
Source: PLoS Pathog. 2021 May 28;17(5):e1009594. doi: 10.1371/journal.ppat.1009594 (PMC8191919; doi:10.1371/journal.ppat.1009594)
Supplement: S5 Table — (DOCX) [file ppat.1009594.s014.docx]

**Table S5. Immunological data for all volunteers in Seattle PfSPZ CVac trial, as measured by PfCSP ELISA, aIFA, and ISI assays.** All out-of-range values and zeroes are reported as “1.00”. * Participant did not proceed to CHMI.

| **Group (PfSPZ-CVac/Dose)** | **Subject ID** | **Infection** | **ELISA (PfCSP OD 1.0)** | | | | | | | | | |
| --- | --- | --- | --- | --- | --- | --- | --- | --- | --- | --- | --- | --- |
|  |  |  | **Pre-Immune** | **Two weeks post third dose** | **Net two weeks post third dose** | **Ratio two weeks post third dose** | **Pre-CHMI** | **Net Pre-CHMI** | **Ratio Pre-CHMI** | **CHMI**  **+28** | **Net CHMI**  **+28** | **Ratio CHMI**  **+28** |
| **Group 1 (5.12 x 10^4^ PfSPZ-CVac administered DVI on Days 3, 10, 17)** | 8 | Infected | 56 | 614 | 558 | 10.96 | 266 | 210 | 4.75 | 1460 | 1404 | 26.07 |
|  | 7 |  | 116 | 1326 | 1210 | 11.43 | 2115 | 1999 | 18.23 | 630 | 514 | 5.43 |
|  | 6 |  | 137 | 14111 | 13974 | 103.00 | 2750 | 2613 | 20.07 | 2777 | 2640 | 20.27 |
|  | 5 |  | 91 | 6750 | 6659 | 74.18 | 3063 | 2972 | 33.66 | 1310 | 1219 | 14.40 |
|  | 4 |  | 81 | 6369 | 6288 | 78.63 | 1760 | 1679 | 21.73 | 2269 | 2188 | 28.01 |
|  | 3 |  | 50 | 334 | 284 | 6.68 | 145 | 95 | 2.90 | 171 | 121 | 3.42 |
|  | 2 |  | 73 | 4153 | 4080 | 56.89 | 1709 | 1636 | 23.41 | 1412 | 1339 | 19.34 |
|  | **Median** | | **81** | **4153** | **4080** | **56.89** | **1760** | **1679** | **20.07** | **1412** | **1339** | **19.34** |
| **Group 2 (1.024 x 10^5^ PfSPZ-CVac administered DVI on Days 3, 10, 17)** | 10 | No CHMI | 64 | 3825 | 3761 | 59.77 | - | - | - | - | - | - |
|  | 11 |  | 1 | 2644 | 2643 | 2644.00 | - | - | - | - | - | - |
|  | **Median** | | **33** | **3235** | **3202** | **1351.88** |  |  |  |  |  |  |
| **Group 3 (1.024 x 10^5^ PfSPZ-CVac administered DVI on Days 1, 6, 11)** | 12 | **Uninfected** | 1 | 729 | 728 | 729.00 | 465 | 464 | 465.00 | 1486 | 1485 | 1486.00 |
|  | 16 |  | 38 | 2207 | 2169 | 58.08 | 1300 | 1262 | 34.21 | 604 | 566 | 15.89 |
|  | 17 |  | 1 | 11713 | 11712 | 11713.00 | 8043 | 8042 | 8043.00 | 4536 | 4535 | 4536.00 |
|  | 18 |  | 55 | 6260 | 6205 | 113.82 | 1441 | 1386 | 26.20 | 984 | 929 | 17.89 |
|  | 19 |  | 96 | 3409 | 3313 | 35.51 | 2247 | 2151 | 23.41 | 1571 | 1475 | 16.36 |
|  | 20 |  | 21 | 8904 | 8883 | 424.00 | 3850 | 3829 | 183.33 | 2173 | 2152 | 103.48 |
|  | **Median** | | **30** | **4835** | **4759** | **268.91** | **1844** | **1769** | **108.77** | **1529** | **1480** | **60.68** |
|  | 13 | **Infected** | 28 | 17183 | 17155 | 613.68 | 2737 | 2709 | 97.75 | 2030 | 2002 | 72.50 |
|  | 15 |  | 12 | 2648 | 2636 | 220.67 | 208 | 196 | 17.33 | 575 | 563 | 47.92 |
|  | **Median** | | **20** | **9916** | **9896** | **417.17** | **1473** | **1453** | **57.54** | **1303** | **1283** | **60.21** |
|  | **Group Median** | | **25** | **4835** | **4759** | **322.33** | **1844** | **1769** | **65.98** | **1529** | **1480** | **60.21** |
| **Saline placebo administered DVI on Days 1,6,11** | 21 | Infected | 73 | 88 | 15 | 1.21 | 1 | 1 | 0.01 | 286 | 213 | 3.92 |
|  | 22 |  | 80 | 103 | 23 | 1.29 | 56 | 1 | 0.70 | 73 | 1 | 0.91 |
|  | 23 |  | 89 | 1 | 1 | 0.01 | 109 | 20 | 1.22 | 197 | 108 | 2.21 |
|  | 24* |  | 82 | 84 | 2 | 1.02 | - | - | - | - | - | - |
|  | **Median** | | **81** | **86** | **9** | **1.11** | **56** | **1** | **0.70** | **197** | **108** | **2.21** |
| **Group (PfSPZ-CVac/Dose)** | **Subject ID** | **Infection** | **aIFA (AFU 2x10^5^)** | | | | | | | | | |
|  |  |  | **Pre-Immune** | **Two weeks post third dose** | **Net two weeks post third dose** | **Ratio two weeks post third dose** | **Pre-CHMI** | **Net Pre-CHMI** | **Ratio Pre-CHMI** | **CHMI**  **+28** | **Net CHMI**  **+28** | **Ratio CHMI**  **+28** |
| **Group 1 (5.12 x 10^4^ PfSPZ-CVac administered DVI on Days 3, 10, 17)** | 8 | Infected | 41 | 1331 | 1290 | 32.46 | 565 | 524 | 13.78 | 654 | 613 | 15.95 |
|  | 7 |  | 1444 | 6509 | 5065 | 4.51 | 919 | 1 | 0.64 | 1430 | 1 | 0.99 |
|  | 6 |  | 77 | 1839 | 1762 | 23.88 | 2536 | 2459 | 32.94 | 6494 | 6417 | 84.34 |
|  | 5 |  | 62 | 29079 | 29017 | 469.02 | 2943 | 2881 | 47.47 | 2951 | 2889 | 47.60 |
|  | 4 |  | 51 | 15027 | 14976 | 294.65 | 1960 | 1909 | 38.43 | 1607 | 1556 | 31.51 |
|  | 3 |  | 510 | 1419 | 909 | 2.78 | 499 | 1 | 0.98 | 879 | 369 | 1.72 |
|  | 2 |  | 298 | 20376 | 20078 | 68.38 | 2872 | 2574 | 9.64 | 2936 | 2638 | 9.85 |
|  | ***Median*** | | ***77*** | ***6509*** | ***5065*** | ***32.46*** | ***1960*** | ***1909*** | ***13.78*** | ***1607*** | ***1556*** | ***15.95*** |
| **Group 2 (1.024 x 10^5^ PfSPZ-CVac administered DVI on Days 3, 10, 17)** | 10 | No CHMI | 73 | 7009 | 6936 | 96.01 | - | - | - | - | - | - |
|  | 11 |  | 4 | 16823 | 16819 | 4205.75 | - | - | - | - | - | - |
|  | ***Median*** | | **39** | **11916** | **11878** | **2150.88** |  |  |  |  |  |  |
| **Group 3 (1.024 x 10^5^ PfSPZ-CVac administered DVI on Days 1, 6, 11)** | 12 | **Uninfected** | 54 | 6549 | 6495 | 121.28 | 1151 | 1097 | 21.31 | 644 | 590 | 11.93 |
|  | 16 |  | 1993 | 15997 | 14004 | 8.03 | 2642 | 649 | 1.33 | 1028 | 1 | 0.52 |
|  | 17 |  | 311 | 23183 | 22872 | 74.54 | 13303 | 12992 | 42.77 | 5921 | 5610 | 19.04 |
|  | 18 |  | 212 | 46247 | 46035 | 218.15 | 24516 | 24304 | 115.64 | 1799 | 1587 | 8.49 |
|  | 19 |  | 1874 | 12066 | 10192 | 6.44 | 4392 | 2518 | 2.34 | 2397 | 523 | 1.28 |
|  | 20 |  | 68 | 21698 | 21630 | 319.09 | 4212 | 4144 | 61.94 | 3523 | 3455 | 51.81 |
|  | **Median** | | **262** | **18848** | **17817** | **97.91** | **4302** | **3331** | **32.04** | **2098** | **1089** | **10.21** |
|  | 13 | **Infected** | 77 | 72659 | 72582 | 943.62 | 3260 | 3183 | 42.34 | 2210 | 2133 | 28.70 |
|  | 15 |  | 863 | 11014 | 10151 | 12.76 | 1394 | 531 | 1.62 | 1279 | 416 | 1.48 |
|  | **Median** | | **470** | **41837** | **41367** | **478.19** | **2327** | **1857** | **21.98** | **1745** | **1** | **15.09** |
|  | **Group Median** | | **262** | **18848** | **17817** | **97.91** | **3736** | **2851** | **31.83** | **2005** | **1089** | **10.21** |
| **Saline placebo administered DVI on Days 1,6,11** | 21 | Infected | 572 | 88 | 1 | 0.15 | 31 | 1 | 0.05 | 130 | 1 | 0.23 |
|  | 22 |  | 92 | 53 | 1 | 0.58 | 741 | 649 | 8.05 | 1314 | 1222 | 14.28 |
|  | 23 |  | 1893 | 40 | 1 | 0.02 | 257 | 1 | 0.14 | 172 | 1 | 0.09 |
|  | 24* |  | 178 | 280 | 102 | 1.57 | - | - | - | - | - | - |
|  | **Median** | | **375** | **71** | **1** | **0.36** | **257** | **1** | **0.14** | **172** | **1** | **0.23** |
| **Group (PfSPZ-CVac/Dose)** | **Subject ID** | **Infection** | **ISI (Reciprocal serum dilution for 80% inhibition)** | | | | | | | | | |
|  |  |  | **Pre-Immune** | **Two weeks post third dose** | **Net two weeks post third dose** | **Ratio two weeks post third dose** | **Pre-CHMI** | **Net Pre-CHMI** | **Ratio Pre-CHMI** | **CHMI**  **+28** | **Net CHMI**  **+28** | **Ratio CHMI**  **+28** |
| **Group 1 (5.12 x 10^4^ PfSPZ-CVac administered DVI on Days 3, 10, 17)** | 8 | **Infected** | 8.97 | 34.61 | 25.64 | 3.86 | 39.32 | 30.35 | 4.38 | 36.91 | 27.94 | 4.11 |
|  | 7 |  | 4.53 | 115.50 | 110.97 | 25.50 | 65.91 | 61.38 | 14.55 | 78.30 | 73.77 | 17.28 |
|  | 6 |  | 1.00 | 75.26 | 74.26 | 75.26 | 23.87 | 22.87 | 23.87 | 46.73 | 45.73 | 46.73 |
|  | 5 |  | 6.28 | 32.21 | 25.93 | 5.13 | 33.45 | 27.17 | 5.33 | 26.73 | 20.45 | 4.26 |
|  | 4 |  | 2.92 | 22.92 | 20.00 | 7.85 | 19.39 | 16.47 | 6.64 | 52.53 | 49.61 | 17.99 |
|  | 3 |  | 4.11 | 26.06 | 21.95 | 6.34 | 1.87 | 1.00 | 0.45 | 1.00 | 1.00 | 0.24 |
|  | 2 |  | 5.43 | 20.11 | 14.68 | 3.70 | 34.18 | 28.75 | 6.29 | 33.53 | 28.10 | 6.17 |
|  | **Median** | | **4.53** | **32.21** | **25.64** | **6.34** | **33.45** | **27.17** | **6.29** | **36.91** | **28.10** | **6.17** |
| **Group 2 (1.024 x 10^5^ PfSPZ-CVac administered DVI on Days 3, 10, 17)** | 10 | **No CHMI** | 5.52 | 58.89 | 53.37 | 10.67 | - | - | - | - | - | - |
|  | 11 |  | 7.99 | 24.19 | 16.20 | 3.03 | - | - | - | - | - | - |
|  | **Median** | | **6.76** | **41.54** | **34.79** | **6.85** |  |  |  |  |  |  |
| **Group 3 (1.024 x 10^5^ PfSPZ-CVac administered DVI on Days 1, 6, 11)** | 17 | Uninfected | 1.00 | 97.86 | 96.86 | 97.86 | 29.91 | 28.91 | 29.91 | 33.99 | 32.99 | 33.99 |
|  | 18 |  | 4.21 | 50.85 | 46.64 | 12.08 | 46.00 | 41.79 | 10.93 | 15.45 | 11.24 | 3.67 |
|  | 19 |  | 15.93 | 28.63 | 12.70 | 1.80 | 16.92 | 0.99 | 1.06 | 11.94 | 1.00 | 0.75 |
|  | 20 |  | 1.00 | 69.35 | 68.35 | 69.35 | 25.67 | 24.67 | 25.67 | 28.49 | 27.49 | 28.49 |
|  | 12 |  | 8.79 | 42.35 | 33.56 | 4.82 | 62.15 | 53.36 | 7.07 | 23.19 | 14.40 | 2.64 |
|  | 16 |  | 6.98 | 20.84 | 13.86 | 2.99 | 16.24 | 9.26 | 2.33 | 62.57 | 55.59 | 8.96 |
|  | **Median** | |  |  |  |  |  |  |  |  |  |  |
|  | 13 | Infected | 6.57 | 80.36 | 73.79 | 12.23 | 38.54 | 31.97 | 5.87 | 35.40 | 28.83 | 5.39 |
|  | 15 |  | 9.89 | 55.26 | 45.37 | 5.59 | 26.35 | 16.46 | 2.66 | 30.11 | 20.22 | 3.04 |
|  | **Median** | |  |  |  |  |  |  |  |  |  |  |
|  | **Group Median** | | **6.78** | **53.06** | **46.01** | **8.83** | **28.13** | **26.79** | **6.47** | **29.30** | **23.86** | **4.53** |
| **Saline placebo administered DVI on Days 1,6,11** | 21 | **Infected** | 1.00 | 1.00 | 1.00 | 1.00 | 13.02 | 12.02 | 13.02 | 31.15 | 30.15 | 31.15 |
|  | 22 |  | 11.71 | 19.35 | 7.64 | 1.65 | 1.00 | 1.00 | 0.09 | 14.12 | 2.41 | 1.21 |
|  | 23 |  | 1.00 | 8.32 | 7.32 | 8.32 | 6.55 | 5.55 | 6.55 | 15.95 | 14.95 | 15.95 |
|  | 24* |  | 13.51 | 1.00 | 1.00 | 0.07 | - | - | - | - | - | - |
|  | **Median** | | **6.36** | **4.66** | **4.16** | **1.33** | **6.55** | **5.55** | **6.55** | **15.95** | **14.95** | **15.95** |
