# Supplementary material for: PfSPZ-CVac efficacy against malaria increases from 0% to 75% when administered in the absence of erythrocyte stage parasitemia: A randomized, placebo-controlled trial with controlled human malaria infection
Source: PLoS Pathog. 2021 May 28;17(5):e1009594. doi: 10.1371/journal.ppat.1009594 (PMC8191919; doi:10.1371/journal.ppat.1009594)
Supplement: S1 Text — (DOCX) [file ppat.1009594.s015.docx]

**S1 Text.**

**Additional information about study randomization**

Once consented, subjects were enrolled by entry of demographic data and confirmation of eligibility for the trial. Within each of the three dose groups, subjects were randomly assigned to receive the study test product or placebo in a 9:3 ratio. Subjects received the same treatment for all three administrations of study product by DVI. Enrollment of subjects was done online using the enrollment module of AdvantageEDC. The randomization code was prepared by statisticians at the Statistical and Data Coordinating Center and included in the enrollment module for the trial. AdvantageEDC generated a treatment assignment for each subject after the demographic and eligibility data had been entered into the system. A designated individual was provided with a code list for emergency unblinding purposes, which was kept in a secure place. Randomization was performed within each cohort, with no other stratification. To ensure balance in the case the trial was halted early, blocks of size four were used. To minimize the chance of modifying the treatment assignment table for unexpected circumstances, three replacement assignments were generated for each cohort: two each of treatment and one each of placebo.

**Additional information about study blinding**

For Study Groups 1 and 2, subjects, investigators, and study staff other than unblinded pharmacist (or other unblinded personnel involved in the preparation of study product) were blinded to the subject's treatment assignment (PfSPZ Challenge vs. placebo). Laboratory personnel performing antibody assays, qRT-PCR, and other assays were blinded to treatment assignment. The clinical and regulatory teams from Sanaria not involved in the preparation of study product were likewise blinded to treatment assignment.

The unblinded site research pharmacist prepared the study product per the randomization assignment. Once prepared, the syringe containing the study product was handed to the clinical team for injection, without revealing the treatment allocation. The study product was

administered by DVI by a blinded administrator. The study vaccine and the saline placebo were identical in appearance; therefore, neither the blinded administrator nor the subject could be unblinded by examining the syringe or its contents.

The qRT-PCR results for samples obtained during the vaccination phase were reported to the study investigators in a blinded fashion during the vaccination phase. In the reports routinely distributed by the qRT-PCR lab to the investigators during the vaccination phase, each test result was identified only by a randomly generated unique specimen ID, which did not link to the individual subject study ID and did not link to prior or subsequent samples obtained from the individual subject. Designated unblinded laboratory staff and study staff maintained test reports that linked the qRT-PCR results to the subject study ID. Staff who maintained the results that were unblinded to qRT-PCR results by subject ID were not involved in any study subject assessments. After the vaccination phase, the vaccination phase qRT-PCR results may have been unblended to some investigators. The qRT-PCR results for samples obtained during the CHMI phase were not blinded. TBS testing was not routinely performed and therefore it was not possible to blind those results when the test was performed for an individual subject. Group 3 was administered the study product open-label.
